# Valorisation Potential of Using Organic Side Streams as Feed for *Tenebrio molitor*, *Acheta domesticus* and *Locusta migratoria*

**DOI:** 10.3390/insects12090796

**Published:** 2021-09-05

**Authors:** Meggie Van Peer, Lotte Frooninckx, Carl Coudron, Siebe Berrens, Carlos Álvarez, David Deruytter, Geert Verheyen, Sabine Van Miert

**Affiliations:** 1 Radius, Thomas More University of Applied Sciences, Kleinhoefstraat 4, 2440 Geel, Belgium; meggie.vanpeer@thomasmore.be (M.V.P.); lotte.frooninckx@thomasmore.be (L.F.); siebe.berrens@thomasmore.be (S.B.); sabine.vanmiert@thomasmore.be (S.V.M.); 2 Provincial Research and Advice Centre for Agriculture and Horticulture, 8800 Rumbeke-Beitem, Belgium; carl.coudron@inagro.be (C.C.); david.deruytter@inagro.be (D.D.); 3 Teagasc Food Research Centre, Department of Food Quality and Sensory Science, D15 KN3K Dublin, Ireland; carlos.alvarez@teagasc.ie

**Keywords:** insect production, biomass, valorisation, side streams, by-products

## Abstract

**Simple Summary:**

The demand for more sustainable protein sources is growing. Insects are believed to have potential as a sustainable protein source since they can be produced with lower environmental impact than current livestock. Furthermore, they have proven to be able to convert low-nutrient biomass into own nutrient-rich body mass, making them nutritionally interesting for applications such as food, feed and technical applications. The aim of this review was to provide more insight on the valorisation potential of agricultural side streams in feed for the rearing of yellow mealworm (*Tenebrio molitor)*, house cricket (*Acheta domesticus)* and migratory locust (*Locusta migratoria)*. Several key aspects need to be considered when aiming to valorise side streams in insect feed. The following aspects are discussed in detail: European legislation, the insects’ dietary requirements, the nutritional composition of insects and the availability of agricultural side streams in the EU. Additionally, research focusing on the production of insects on side streams is reviewed. Through this review, it is concluded that there is a huge potential to valorise residues in feed for the insects of interest. However, it is not a self-evident process.

**Abstract:**

Due to increasing welfare and population, the demand for alternative protein sources, obtained with minimal use of natural resources, is rising in today’s society. Insects have the potential to be used as an alternative protein source since they are considered to be able to convert low-value biomass into high-value components, resulting in opportunities for valorisation of organic side streams. Moreover, insects are suggested to be a sustainable protein source, referring to the efficient “feed to body” mass conversion potential. The aim of this review was to explore the potential to rear the yellow mealworm (*Tenebrio molitor*), the house cricket (*Acheta domesticus*) and the migratory locust (*Locusta migratoria*) on low or not yet valorised organic side streams within the food supply chain. This was performed by collecting research information focusing on the rearing of the insects in scope on organic biomass. In addition, the nutritional composition of the produced insects as well as their dietary requirements will be reviewed. Finally, the availability of side streams in the EU will be discussed as well as their potential to be used as insects feed.

## 1. Introduction

Today’s society is facing enormous challenges. While some countries are experiencing unprecedented growing rates, other countries are facing a decline in population growth along with the ageing of the population. In both cases, the need for more protein production is capital: In the first scenario, there is a need to cope with a higher demand for food amounts; and in the second one there is a need to prevent some diseases related to the lack of protein in elderly population diets, such as sarcopenia [1]. The global population is growing and is expected to reach 9.7 billion people by 2050 [2]. Associated with this growth and the rising prosperity, the demand for food is estimated to increase by 70–80% between 2012 and 2050 [3,4]. Moreover, high amounts of biomass related to the production and consumption of food is lost or wasted, lowering the sustainability of our food systems. According to the Food and Agriculture Organisation of the United Nations (FAO), roughly one-third of the global food production for human consumption (ca. 1.3 billion tons per year) is lost or wasted [5] of which 88 Mt in the EU [6]. Some of the produced side streams have already been valorised in animal feed applications; however, their use as feedstuffs remains limited (4%) [6,7]. Furthermore, current food production and consumption have a profound impact on climate change and depletion of natural resources. For example, current livestock farming is responsible for about 15% of the total greenhouse gas emissions caused by humans [3,8]. Consequently, the demand for alternative and more sustainable protein sources is rising.

Several innovative solutions are continuously being proposed and investigated for tackling the challenges that global society is facing, either to implement healthier diets or to provide the minimum protein intake required. The sustainable use of biomass is expected to become an increasingly important factor here. Several attempts have been made to clearly define biomass side streams. However, it is often not clear what is meant by the terms used. Agricultural side streams and residues are often defined as food waste. In order to be able to account food waste from the food supply chain, it is imperative to properly define what is meant by this term. In this review, the definition of food waste as defined by the FUSIONS definitional framework is used, i.e., “food waste is any food, and inedible parts of food, removed from the food supply chain to be recovered or disposed (including composted, crops ploughed in/not harvested, anaerobic digestion, bio-energy production, co-generation, incineration, disposal to sewer, landfill or dis-carded to sea)”. The food supply chain is the connected series of activities used to produce, process, distribute and consume food. It consists of different sectors where the losses or wastes can be produced, namely at primary production level, processing and manufacturing level, at wholesale and retail level and at the level of consumption (households) [9].

Since insects have proven to be able to efficiently convert low-value biomass into their own biomass consisting of high-quality components, i.e., proteins, fats and chitin [10,11,12], they have potential to tackle the above-described societal challenges [13,14], as was already indicated in 1975 by Meyer-Rochow [15]. Using insect production to valorise side streams is a strategy that has gained increased attention. Insects are believed to have a lower ecological impact than current livestock. Moreover, research has shown that insects produce less greenhouse gases and emit significantly less ammonia [16]. In addition, insects convert feed into biomass more efficiently than conventional livestock [14] because they are ectothermic and, therefore, use little energy to maintain their body temperature [17]. Furthermore, insects can be produced in vertical farming systems, making them more productive per m^2^ [18], and they generally require less water [19,20]. Insects, therefore, might provide an attractive key in the circular economy, meeting the requirements of a more sustainable society.

The aim of this review is to provide more insight on the potential of using agricultural side streams as feed for the production of the yellow mealworm, the house cricket and the migratory locust. This review is focused on these three species since they are the main ones already being sold as food in the EU, and they are currently approved for food applications (mealworm and migratory locust) or submitted for approval within EU legislation. Although it is expected that other species will be included in the future, its evaluation by EFSA is still pending, and this is a substantial barrier for the use of other insect species until a final opinion is released. Among the species under EFSA consideration, *Hermetia illucens* (black soldier fly) (BSF) is also included, which is currently under evaluation as per the Novel Food authorisation list (https://ec.europa.eu/food/safety/novel-food/authorisations/summary-applications-and-notifications_en) (accessed on 30 July 2021).

Although BSF is also commercially reared, this species is considered out of scope for this review as it is mainly investigated as feed in poultry, fish and swine [21]. Several dossiers for insects have been submitted, including species of mealworms, grasshoppers and house crickets, from which EFSA has released a favorable opinion for mealworms and migratory locusts in 2021 [22,23]. In addition to general food hygiene requirements, the productions and marketing of insects as food are regulated by the now repealed EU Novel Foods Regulation of 1997. Under this law, foods that were not consumed to a significant degree in Europe before 1997 may only be placed on the market if specific authorization has been granted, following a positive safety assessment. The vagueness regarding insect products was tackled by the 2015 revision of the legislation. It confirmed that insects are indeed novel foods and harmonized authorization requirements across the EU. Producers of insect-based foods can now obtain EU-wide authorization to produce and sell their products. Some countries only allowed certain species to be produced and sold as food and some authorized all species, while other countries completely prohibited the production and marketing insect-based products as novel foods. Since the absence of insect species on the 1997 list constituted a de facto ban on insect-based foods, transitional measures were included in the Regulation. This allowed existing producers to continue marketing their insect-based foods in member states where it had already been authorized under the 1997 Regulation. This explains why some insect-based food products are already sold in Belgium, United Kingdom, France, Germany, the Netherlands, Denmark and Finland—ahead of their EU-level authorization. A more detailed description on the EU regulatory framework for insects as food and feed has been published by Montanari and co-authors [24].

Different points to be taken into account when rearing insects on side streams are highlighted and translated in challenges and opportunities for the upcoming insect sector. First, an overview of the side streams that have already been investigated as feed for the insect species in scope is given. In addition, the influences of the nutritional quantity and quality of insect feed on growth and composition are discussed. Finally, the availability of low or not yet valorised side streams in the EU is explored.

## 2. Insect Production on Organic Side Streams

Insects are considered efficient in biomass utilization for own mass gain. Depending on the insect species and their diet, they have proven to contain high amounts of high-quality components such as proteins, fat and chitin. They can contain over 70% of proteins and fat (dry matter basis) [25,26,27]. Therefore, insect rearing provides an attractive pathway to be used in the circular economy. Insect bioconversion allows the recapture of nutrient losses in the food supply chain [28,29]. Moreover, the produced insects’ residue has proven potential application as organic fertilizer [30].

Currently, reared insects are mostly fed with diets that compete with the human food and animal feed production. Using low value by-products to feed the insects solves this problem; creates opportunities to valorise products that are now mostly fermented, composted or discarded; and might potentially reduce the cost of insect rearing practices [28,29].

Since insect rearing seems to be a promising approach for a sustainable use of biomass, this recently gained the interest of various researchers worldwide. Several studies have already focused on the use of organic side streams for the rearing of insects. Studies focusing on the insect species in scope are discussed below.

### 2.1. Vegetable By-Products in Insects Diet

#### 2.1.1. *Tenebrio molitor*

*T. molitor* is a cosmopolitan insect species, but it also naturally occurs in the north temperate regions of Europe. Nowadays, they are distributed worldwide. The larvae feed on stored grain products, which is the reason why they are considered a pest insect. They also feed on animal material, e.g., meat scraps, dead insects and feathers [31]. These natural feeding habits open up possibilities to rear the mealworms on side streams.

Below, studies investigating the use of vegetable side streams in feed for the rearing of *T. molitor* are discussed. An overview of the chemical composition of the diets used in these studies is given in Table 1.

Van Broekhoven et al. [11] investigated larval growth and survival of three edible mealworm species when grown on diets composed of organic by-products. They used four mixtures of maize DDGS (Distillers Dried Grains and Solubles), beer yeast, bread remains and potato peelings. The four tested diets contained different amounts of these ingredients and differed with respect to protein and starch content, i.e., high in both protein (24.1%) and starch (28.4%) (HPHS), high in protein (32.5%) and low in starch (7.4%) (HPLS), low in protein (10.7%) and high in starch (49.8%) (LPHS) and low in both protein (20%) and starch (19.4%) (LPLS). Two diets used by commercial mealworm producers were used as control diets, composed of 18.8% crude protein and 43.6% starch for control diet A and 15.5% crude protein and 23.0% starch for control diet B. Carrots were added to all diets to provide moisture. Larvae were allowed to feed ad libitum. Remarkably, the HS diets were initially mixed with cookie remains but replaced by potato steam peelings as the original mixture resulted in total mortality. It was suggested that the spices in the cookies (especially cinnamon) could be toxic for the mealworms. For *T. molitor*, survival on all diets was similar to control diet B and significantly better compared to control diet A. Development time of *T. molitor* was shorter on the HPHS, HPLS and LPLS diets compared to control diets and longest on the LPHS diet. Pupal weight of *T. molitor* was more on the HPLS and less on the LPHS diet compared to the control diets. The LPHS also had the lowest feed conversion ratio (on a fresh matter base, calculated as weight of ingested food/weight gained). It was suggested that the LPHS diet might lack nutrients or contained compounds which are harder to digest or toxic to mealworms, referring to the high amount of potato steam peelings used in this diet (85%). This could be explained by the digestion resistance of potato starch by Tenebrionidae, which is higher than starch from wheat or maize [32,33]. In addition, potato glycoalkaloids, which persist after processing [34] can have a toxic effect on insects that do not naturally consume potato [35,36]. However, potato steam peelings did not seem to be as toxic to *T. molitor* as for Zophobas atratus, which had a much lower survival rate (27%) on the LPHS [11].

Oonincx et al. [12] investigated, among others, a mixture of freeze-dried side streams (spent grains, beer yeast and cookie remains) that supported good growth of *T. molitor*. Four diets were formulated defined by varying protein and fat content, i.e., high protein (21.9%), high fat (9.5%) (HPHF), high protein (22.9%), low fat (1%) (HPLF), low protein (12.9%), high fat (14.6%) (LPHF) and low protein (14.4%), low fat (2.1%) (LPLF). Two diets used by commercial mealworm producers were used as control diets, composed of 17.5% crude protein and 4.9% fat for control diet 1 and 17% crude protein and 4.2% fat for control diet 2. Carrots were provided as a moisture source. *T. molitor* developed fastest on the high protein diets. Survival was similar to control diets except for the LPHF diet, which had increased mortality. This could be due to the high inclusion of the cookie remains, which also caused mortality in the study of van Broekhoven et al. [11]. Compared to the control diets, feed conversion efficiency for the high protein diets was similar, but significant higher for the low protein diets.

Furthermore, Ramos-Elorduy et al. [37] successfully reared yellow mealworms on side streams. In their study, five diets composed of a mixture of organic waste (freeze-dried cereals, fruit and vegetables), yeast and beetle excreta were investigated. The diets were balanced to consist of approximately 20% protein. As a control diet, a mixture of wheat bran (90%) and yeast (10%), which consists of 22.46% protein, was used. Based on total biomass produced, diet E (17.63% protein, 14.92% fat and 61.17% carbohydrates) had the highest quantity, whereas diet A (12.05% protein, 16.56% fat and 62.64% carbohydrates) had the lowest yield. Survival (numbers of harvested larvae) and individual weight were also different on the diets. Survival between samples was ordered as follows: diet D > diet B > diet C > diet E > diet A. Typically, the lower the number of larvae, the higher the weight. Since the nutritional content is fairly similar for the diets investigated, differences were probably caused by differences in nutritional quality and physical properties of the waste streams.

Stull et al. [38] investigated whether *T. molitor* could be reared on agricultural by-products from maize production. Larvae were grown on a mixed soy, maize grain and stover diet and a 100% stover diet. While all diets were accepted by *T. molitor* larvae, final weight of the mixed diet was only 70% compared to the control diet; for the 100% stover diet, this even dropped to less than 50%.

Mancini et al. [39] investigated the use of brewery spent grains, bread and cookies in the diet of *T. molitor*. Mealworms grown on diets composed of spent grains (SG) or spent grain and cookie mixture (SG-C) larvae performed better than diets composed of cookies (C), bread (B) and of a mixture thereof (B-C). The authors concluded that the dietary efficiency of the mealworms was related to the chemical composition of the diet. Diets containing the highest protein content (SG; SG-C), showed the lowest FCR values (2.22, 2.76 on fresh weight basis). Larvae fed with diets B, C and the mix B-C showed high FCR values: about 8.86, 7.31 and 4.02. Varying FCR values related to protein content of the diet are also reported by other authors [11,12].

In a recent study [40], the effects of vegetable waste, garden waste, cattle manure and horse manure on growth and survival rates and nutritional value of *T. molitor* were investigated. The side streams were chopped to 2–4 cm long pieces, and manures were broken into small pieces. The diets were formulated with 90% of each side streams mixed with 10% chickenfeed, which was also used as a control diet. The larvae were fed ad libitum throughout the experimental period. Despite the low macronutrient content, larvae grown on the diets composed of garden waste (7.618% protein, 1.401% carbohydrates and 0.424% fat) or vegetable waste (2.788% protein, 1.259% carbohydrates and 0.234% fat) were only slightly smaller than those grown on pure chickenfeed. Even the manures supported a steady growth.

In the study of Li et al. [27], yellow mealworms were reared on inedible parts of wheat (straw) and vegetables (old leaves). The straw was fermented using three different techniques (facultative anaerobic fermentation, aerobic fermentation and yeast fermentation) to improve the quality of the feed, i.e., improvement of digestibility and protein contents. The mealworms reared on the plant wastes reached a fresh end weight of 56.15% (dry 46.76%) of the larvae fed on the control diet (wheat bran). The growth curve showed a reduced growth rate compared to the control at the last 15 days. This was possibly due to the lack of nitrogen in the fermented straw (0.6% w/w) compared to wheat bran (2.6%) [41]. The authors concluded that growth of larvae requires abundant nitrogen in order to synthesize proteins.

Zhang et al. [26] studied the potential of three agri-food industry by-products (mushroom spent corn stover (MSCS), highly denatured soybean meal (HDSM) and spirit distillers’ grains (SDG) as feed for *T. molitor*. Mealworms dry end weights reared on three by-products were 53–67% of the control (wheat bran). Compared to the control, slower growth rates for three treatments were observed for the last 20 days of the larval phase. However, Zhang et al. suggested some approaches that can be taken to maintain the growth rate for the last 20 days. Slower growth rate with MSCS treatment was probably due to the low protein content (4% compared to 17% in wheat bran) in the diet [27], as larvae tend to eat less and gain less weight on nutrient imbalanced diet [42]. Protein addition to the diet was found to significantly shorten development time and improve food conversion efficiency [43] and is, therefore, suggested at around 40 days with MSCS to maintain fast growth. Slower growth rates with HDSM and SDG treatments were probably due to protein denaturation during their distillation process. Zhang et al. also suggested that vitamin supplementation to all three diets should be explored in the future to increase growth rate and reduce mortality. Feed conversion and utilization ratios ranged from 36% to 45% and 50% to 57% of control, respectively.

In the study of Melis et al. [44], the rearing of *T. molitor* on dried brewers’ spent grains (BSG) was investigated and compared to the conventional feed, which was wheat bran. Mealworms reared on BSG showed a lower feed conversion ratio (4.352 ± 0.451; (weight of ingested feed/mealworm weight gained) × 100 and expressed on a dry matter basis) compared to the control (5.48 ± 0.197). The average of larval end weight per tray was 1624.4 g (control) and 1599.5 g (BSG), respectively, while the average of ingested feed per tray was 3550 g and 2963 g for the control and BSG, respectively. No differences in development time were found between larvae reared on the control or BSG diet (14.10 and 14.25 weeks, respectively).

The potential of using low-value organic by-products as feed for yellow mealworms was also explored by Ruschioni et al. [45]. Three pomace-enriched substrates from the olive oil industry (mixtures middlings/pomace 3:1, 1:1 and 1:3) were composed and compared with two control diets (100% organic wheat flour and 100% wheat middlings). Of all tested mixtures, the diet made up of 25% olive pomace and 75% wheat middlings (3:1) appeared to be the best compromise between growth performance and mealworm nutritional properties. The mean larval development time ranged between 98 (control middlings) and 133 days (75% olive pomace) and was clearly depending on the diets. The shortest values were measured for larvae reared on control feed (middlings) and on feed containing 25% of olive pomace. Further increase in olive pomace percentage increased larval development time. The larval end weight ranged from 70 to 131 mg with significantly higher results for larvae fed on control substrates and on 25% pomace enriched feed.

Agricultural residues (leek foliage, cauliflower leaves, Belgian endive roots and Belgian endive white leaves) were tested as wet feed for the production of mealworms in the Interreg 2 Seas project ‘BioBoost’ (2016–2020). During the study, the residues were mixed into a pulp. Similar total mealworm end weights were obtained on the different side streams as on the control wet feed (carrot). The results indicated that moist residues can be used as mealworm wet feed, as long as they are properly mixed. Mixed cauliflower leaves and mixed Belgian endive roots even outperformed carrots, which are often used as moisture source for rearing mealworms [46]. Moreover, in the PDPO project (2017–2019) ‘Witloofwortels, ook meelwormen lusten er wel pap van’, the potential of forced chicory roots and chicory leaves, compared to carrots, as a wet feed for mealworms was investigated. No significant differences between wet feed sources were found [47].

What is important to note is that crop residues are often difficult to digest by farm animals because they contain 30–45% cellulose, 10–40% hemicellulose and 5–25% lignin (% DM) [48] and high quantities of lignocellulose. In addition, crop residues require harsher pre-treatment [49]. This must be considered when rearing insects on cellulose-rich agricultural residues. The use of crop waste residues as supplementary feed for mealworms has been studied by Yang et al. [50]. Five lignocellulose-rich crop residues (wheat straw, rice straw, rice bran, rice husk and corn straw) were tested as potential feed for 6–7 instar mealworms. All residues were pretreated (cut into small pieces; washed; dried in a forced-air drying oven at 45 °C for 48 h; stored in clean polyethene bags at 4 °C). Mealworms were divided into six groups, each fed with wheat bran (control) and one of the five crop residues. Over a 32 days period, 10.0 g of each treatment was supplemented every four days. Yang et al. concluded that mealworms can survive on the tested lignocellulose-rich crop residues as sole feedstock as well as they can when fed with the control over a 32 days period. With the exception of wheat straw and rice husk, the residues supported mealworms’ life activity and growth with consumption of the residues by 90% or higher and degraded lignin, hemicellulose and cellulose. Rice straw, rice bran and corn straw were found suitable as supplementary feeds to rear mealworms.

**Table 1:** Chemical composition (% DM) of discussed diets used for yellow mealworm production.

Diet	Ingredients	Moisture (%)	Ash (%)	Fat (%)	Protein (%)	Crude Fibre (%)	Minerals	Carbo (%)	Starch (%)	NDF (%)	ADF (%)	ADL (%)	H-Cellulose (%)	Cellulose (%)	Reference
HPHS—High Protein High Starch	Maize DDGS 10% Beer Yeast 40% Bread remains 10% Potato steam peelings 40%			4.0 ^1^	24.1 ^1^				28.4 ^1^						van Broekhoven et al. (2015) [11]
HPLS—High Protein Low Starch	Maize DDGS 20% Beer Yeast 40% Bread remains 10% Spent grains 30%			7.0 ^1^	32.5 ^1^				7.4 ^1^						
LPHS—Low Protein High Starch	Beer Yeast 5% Bread remains 10% Potato steam peelings 85%			1.8 ^1^	10.7 ^1^				49.8						
LPLS—Low Protein Low Starch	Beer Yeast 10% Bread remains 50% Spent grains 40%			6.2 ^1^	20.0 ^1^				19.4						
HPHF—Hi Protein High Fat	Spent grains 60% Beer Yeast 20% Cookie remains 20%	5.0		9.5	21.9										Oonincx et al. (2015) [12]
HPLF—High Protein Low Fat	Beer Yeast 50% Potato steam peelings 30% Beet molasses 20%	4.9		1.0	22.9										
LPHF—Low Protein High Fat	Cookie remains 50% Bread 50%	10.9		14.6	12.9										
LPLF—Low Protein Low Fat	Potato steam peelings 30% Beet molasses 20% Bread 50%	10.9		2.1	14.4										
Diet A	Organic waste ^2^ 70% Yeast 10% Excreta of *T. molitor* 20%			16.86	12.5	3.88	4.67	62.64							Ramos-Elorduy et al. (2002) [37]
Diet B	Organic waste ^2^ 70% Yeast 5% Excreta of *T. molitor* 25%			18.28	19.18	3.043	7.79	51.38							
Diet C	Organic waste ^2^ 75% Yeast 10% Excreta of *T. molitor* 15%			19.74	16.77	3.02	5.20	52.68							
Diet D	Organic waste ^2^ 75% Yeast 5% Excreta of *T. molitor* 20%			17.26	15.81	9.50	5.23	52.23							
Diet E	Organic waste ^2^ 80% Yeast 5% Excreta of *T. molitor* 15%			14.92	17.63	6.95	7.38	61.17							
	Organic corn meal 30% Organic soy flour 30%Dry stover 40%														Stull et al. (2019) [38]
	Maize stover dry 100%														
SG	Spent grains 100%	5.19	3.43	3.29	17.98 ^3^			70.10							Mancini et al. (2019) [39]
B	Bread 100%	2.91	1.88	0.31	11.15 ^4^			83.75							
C	Cookies 100%	0.01	0.70	10.44	6.55			82.29							
SG-C	Spent grains 50% Cookies 50%	2.96	2.065	6.865	12.265			76.195							
B-C	Bread 50% Cookies 50%	1.46	1.29	5.375	8.85			83.02							
	Chicken feed 10% Vegetable waste 90% (mixed peels of 10% onion, 25% potato, 25% sweet potato, 30% carrot and 10% cucumber)			0.234	2.788			1.925							Harsányi et al. (2020) [40]
	Chicken feed 10% Garden waste 90% (50% Poaceae species and other common weeds, 25% tree leaves and 25% branches (Populus, Salix, Pinus and Corylus species), a mixture of stone fruits and other ornamental plant parts)			0.424	7.618			1.401							
	Chicken feed 10% Cattle manure 90% (45% straw)			0.319	3.965			1.335							
	Chicken feed 10% Horse manure 90% (65% straw)			0.558	5.033			1.690							
	Fermented wheat straw	22.0								30.09		5.51	29.13	30.06	Li et al. (2013) [27]
MSCS	Mushroom spent corn stover	<10	6.91	12.81	3.90			76.38							Zhang et al. (2019) [26]
SDG	Spirit distillers grains	<10	15.04	6.85	13.87			64.24							
HDSM	Highly denaturated soybean meal	<10	11.61	4.76	43.18			40.45							
BSG	Brewers’ spent grain	22.0	3.82	7.30	22.45					57.95	22.94	8	35	15	Melis et al. (2019) [44]
3:1	Middlings (75%) Olive pomace (25%)	21.96	4.76	6.18	15.42	16.77									Ruschioni et al. (2020) [45]
1:1	Middlings (50%) Olive pomace (50%)	37.42	4.88	6.99	14.89	21.58									
1:3	middlings (25%) Olive pomace (75%)	53.16	5.25	7.44	11.76	31.45									

Legend: Carbo: carbohydrates; NDF: neutral detergent fibre; ADF: acid detergent fibre; ADL: acid detergent lignin; H-cellulose: hemicellulose. ^1.^ Values calculated based on available values for organic by-products (www.duyniebeuker.nl, www.groan.nl, accessed on 14 January 2021). ^2.^ Freeze-dried cereals, fruit and vegetables. ^3.^ N to P factor 5.83 was used. ^4.^ N to P factor 5.70 was used.

#### 2.1.2. *Acheta domesticus*

House crickets are generally believed to originate from Europe, Southeast Asia and North Africa. Due to distribution by man, they now occur worldwide [51,52]. They prefer a warm climate (optimum 30–35 °C) [53]. House crickets are omnivorous, feeding on leaves, seeds, fruit, other live or dead insects, etc.

Below, studies investigating the use of vegetable side streams in feed for the rearing of *A. domesticus* are discussed. An overview of the chemical composition of the diets used in these studies is given in Table 2.

The study of Collavo et al. [54] showed that *A. domesticus* has potential to be reared on organic waste. Four different insect diets were composed and compared: (1) a dairy cow diet (DCD); (2) a dairy cow diet supplemented with yeast to increase cricket yield (DCD + Y); (3) an aromatic arboreal diet (AAD) containing false acacia, yeast, basil leaves, sage leaves, hazel leaves and maple leaves; and (4) a human refuse diet (HRD) containing fruits and vegetables, rice and pasta, pork and beef meat, bread, cheese skins and yolk. The best growth rate was obtained with the HRD treatment. The highest cricket yield was obtained on the HRD after 8–9 weeks (0.45 g/cricket), while for the DCD + Y diet it took 9–10 weeks (0.43 g/cricket). Average end weight on the DCD diet was 0.40 g/cricket and 0.35 g/cricket on the AAD diet (after 10 weeks). Crickets reared on the HRD diet also had better efficiency of conversion of ingested food (ECI) compared to the other diets. The poorest insect yield was obtained using the AAB treatment, attributed largely to the fact that many of the crickets were cannibalized, indicating that this diet was not well nutritionally balanced.

In the study of Oonincx et al. [12], several side streams were investigated as feed ingredient for house crickets’ diet. From these ingredients (freeze-dried beet molasses, potato steam peelings, spent grains and beer yeast, bread remains and cookie remains), four experimental diets were composed: (1) high protein, high fat (HPHF); (2) high protein, low fat (HPLF); (3) low protein, high fat (LPHF); and (4) low protein, low fat (LPLF). Oonincx et al. concluded that survival rates were low on all diets, and feed conversion (on fresh weight basis) was inefficient on most diets (range 2.3–6.1). Possibly, the culture was infected by a densovirus. On the HPHF and the control diet the development time of house crickets was 4.5–11.5 weeks, but development was strongly prolonged on the other diets.

The study of Sorjonen et al. [55] concluded that crickets (*A. domesticus* and *Gryllus bimaculatus*) can be successfully reared on feeds composed with by-products of food industry. In their study, 14 experimental diets containing by-products (potato protein, barley mash, barley feed, turnip rape and mix of broad bean and pea) were investigated. Their diets were based on the principle to replace soy-protein (from Patton’s diet No. 16 [56]) with protein from the side streams. The nutrient contents of each side stream-based diet (except potato protein) were repeated with three levels of protein: 30% in high (H), 22% in medium (M) and 15% in low (L). The potato protein diets were high in protein (30.5%), and they included two diets, one where half of the soybean was replaced with potato protein (potato-half) and the other where all of the soybean was replaced with potato protein (potato-all). The by-product feeds were designed to meet the nutritional demand of crickets. Therefore, the survival of *A. domesticus* was relatively high in by-product diets (64–94%). Moreover, many of the tested diets enhanced growth, development and yield compared to the control diets. The overall best by-product diet for *A. domesticus* was medium-protein barley mash. The study of Sorjonen et al. shows that by-products can be used as a protein source for crickets when the diet is in balance with other nutritional components.

Lundy and Parrella [57] reared *A. domesticus* on five different composed diets, four diets containing food waste and crop residues: (1) filtrate from an aerobic enzymatic digestion process that converts grocery store food waste into 90% liquid fertilizer and 10% solids (FW1); (2) minimally-processed, post-consumer food waste (FW2); (3) a 1:1 ratio of wheat and maize silage containing approximately 50% non-grain aboveground biomass (straw) (CR1); (4) a 2:1:1 ratio of poultry manure, wheat straw and rice straw silage (CR2). Poultry feed (PF) was used as control diet. Diets varied much in chemical composition and were fed ad libitum to the crickets. Similar to the crickets reared on the control diet, populations fed the FW1 diet also demonstrated a steady mass gain and grew to a harvestable size. This is in contrast to the FW2, CR1 or CR2 diets, where none of the populations survived to a harvestable size, probably due to the lack of nutrients in those diets. The FCR of crickets reared on the FW1 treatment was slightly higher (1.91) than the FCR of crickets reared on the control (1.47).

In the study of Harsányi et al. [40], *A. domesticus* was reared on mixtures (90% waste, 10% chicken feed) containing vegetable waste, garden waste, cattle manure and horse manure. The organic waste was chopped into small pieces. Chicken feed was used as control diet. No significant differences between diets were found. Crickets reared on garden and vegetable waste showed only a slightly lower weight after 45 days than the control diet.

**Table 2:** Chemical composition (% DM) of discussed diets used for house cricket production.

Diet	Ingredients	Moisture (%)	Ash (%)	Fat (%)	Crude Protein (%)	Crude Fibre (%)	Minerals	Carbo (%)	Starch (%)	NDF (%)	ADF (%)	ADL (%)	H-Cellulose (%)	Cellulose (%)	Reference
DCD—Dairy Cow Diet	Soybean flour Lucern Corn flour Wheat Silage corn Sugar beet														Collavo et al. (2005) [54]
DCD + Y—DCD + yeast	Soybean flour Lucern Corn flour Wheat flour Yeast Silo Sugar beet														
AAD—Aromatic Arboreal Diet	False acacia Yeast Basel leaves Sage leaves Hazel leaves Maple leaves														
HRD—Human Refuse Diet	Fruits and vegetables Rice and pasta Pork and beef meat Bread Cheese skins Yolk														
HPHF—High Protein High Fat	Spent grains 60% Beer yeast 20% Cookie remains 20%	5.0		9.5	21.9										Oonincx et al. (2015) [12]
HPLF—High Protein Low Fat	Beer yeast 50% Potato steam peelings 30% Beet molasses 20%	4.9		1.0	22.9										
LPHF—Low Protein High Fat	Cookie remains 50% Bread 50%	10.9		14.6	12.9										
LPLF—Low Protein Low Fat	Potato steam peelings 30% Beet molasses 20% Bread 50%	10.9		2.1	14.4										
Potato-half	By-product of potato flour production (10%)			4.0	30.5			51.2							Sorjonen et al. (2019) [55]
Potato-all	By-product of potato flour production (20%)			4.1	30.5			52.2							
Barley mash-High	By-product of beer production (29%)			5.7	30.5			50.5							
Barley mash-Medium	By-product of beer production (41%)			6.5	22.5			58.0							
Barley mash-Low	By-product of beer production (20%)			5.4	15.0			66.0							
Barley feed-High	By-product of ethanol production (15%)			5.2	30.0			51.0							
Barley feed-Medium	By-product of ethanol production (44%)			7.4	22.5			58.2							
Barley feed-Low	By-product of ethanol production (31%)			6.5	15.0			66.0							
Broad bean pea-High	By-products of plant protein source (30%)			3.8	30.0			50.4							
Broad bean pea-Medium	By-products of plant protein source (30%)			3.9	22.5			58.2							
Broad bean pea-Low	By-products of plant protein source (13%)			4.2	15.0			66.0							
Turnip rape-High	By-product of rapeseed oil production (23%)			6.3	30.0			48.4							
Turnip rape-Medium	By-product of rapeseed oil production (5%)			5.9	22.5			56.8							
Turnip rape-Low	By-product of rapeseed oil production (7%)			5.0	15.0			66.0							
Food Waste 1			7.2	19.3	28.8 ^1^						24.7				Lundy and Parrella (2015) [57]
Food Waste 2			21.4	15.8	13.8 ^1^						41.6				
Crop Residu 1			6.4	2.8	9.4 ^1^						25.3				
Crop Residu 2			32.8	1.1	8.8 ^1^						50.1				
	Chicken feed 10% Vegetable waste 90% (mixed peels of 10% onion, 25% potato, 25% sweet potato, 30% carrot and 10% cucumber)			0.234	2.788			1.925							Harsányi et al. (2020) [40]
	Chicken feed 10% Garden waste 90% (50% Poaceae species and other common weeds, 25% tree leaves and 25% branches (Populus, Salix, Pinus and Corylus species) and a mixture of stone fruits and other ornamental plant parts)			0.424	7.618			1.401							
	Chicken feed 10% Cattle manure 90% (45% straw)			0.319	3.965			1.335							
	Chicken feed 10% Horse manure 90% (65% straw)			0.558	5.033			1.690							

Legend: Carbo: carbohydrates; NDF: neutral detergent fibre; ADF: acid detergent fibre; ADL: acid detergent lignin; H-cellulose: hemicellulose. ^1.^ Calculated from %N (N to P factor 6.25).

#### 2.1.3. *Locusta migratoria*

*L. migratoria* is known for its ability to form a plague (occur in high numbers), resulting in them being widely regarded as one of the most severe pests worldwide, distributed mostly in warm regions. Swarms can inflict a lot of damage to green vegetation and crops, resulting in disastrous consequences for soil and agriculture. They primarily feed on gramineous plants, such as reed, barnyard grass and elephant grass, and are therefore considered specialists [58,59,60,61].

Unfortunately, no studies investigating the rearing of *L. migratoria* on side streams could been found by the authors. This is probably due to the fact that locusts are specialists regarding feed. It has been reported by Dadd [62,63,64,65,66], who tested artificial diets as feed for *L. migratoria*, that none of the diets tested were as successful as the control diet, i.e., grass.

In the study of Mehrotra et al. [67], *L. migratoria* was fed with cabbage. They concluded that *L. migratoria* reared on only cabbage resulted in poor weight gain. However, *L. migratoria* did consume the cabbage and with higher consumption rate and digestibility compared to *S. gregaria*. It was concluded that a diet containing only cabbage seemed to be nutritional imbalanced for *L. migratoria*.

Bernays et al. [68], reported that *L. migratoria* mainly feeds on grasses, but there are numerous reports of *L. migratoria* feeding on other plants. In Kenya, it was reported that various legumes were seriously damaged when the cultivated pasture grasses were no longer available [69]. Moreover, the insect is also known to eat dicotyledonous plants in the laboratory (refer to, e.g., [70]). Bernays et al. concluded that in many cases, however, non-grasses are only accepted in the absence of grasses.

The above discussed studies indicate that even though *L. migratoria* primarily feeds on grasses, there might be a potential to rear them on agricultural residues. For example, since wheat leaves and corn leaves are host plants of *L. migratoria* [71], they might also be potentially reared on horticultural foliage, an agricultural residue. However, to the best of our knowledge, this potential has not been explored yet.

### 2.2. Animal Co-Products in Insects Diet

The use of animal co-products, including processing streams rich in proteins as substrate for rearing insects has not been widely explored, despite there being potential. Meat co-products and meat processing streams are an excellent source of proteins, minerals, vitamins and lipids, as previously reviewed [72,73]. However, depending on the species (beef, pork or lamb) and the type of co-product, proximate composition might differ remarkably. For example, fat content can be as low as 0.1% in blood (wet weight) and as high as 70% in pork jowl. In the same manner, protein content can range from 15% to 22% (wet weight), although this parameter is less variable than the fat. What is more relevant than overall protein content is how high the fraction of collagen is compared to total protein. This might be of relevance for insects’ substrate formulation because of the particular properties of this protein: low solubility and thermal resistance and excellent as thickener, in water retention and as a texturizer. In this case, skin, pork hands and lungs are a rich source of collagen, while blood, heart or liver possess less collagen content. The main impact of collagen as a nutrient is its amino acid profile: While it is rich in proline, glycine and hydroxyproline, the amount of other amino acids, including the essential ones, is very low. This lack of essential amino acids might have to be compensated by adding other meat coproducts to the mixture, with a better amino acid profile [72,73].

Using animal origin products as protein supplement has been studied for crickets, as reported by Woodring et al. [74], where a 2.5% of fish meal was employed as part of the diet. A research work carried out in 1967 [56] highlighted that domestic crickets (*A. domesticus*) performed much better in terms of growth rate when dry liver was added to their diet; it was suggested that a growth factor (likely riboflavin, niacin or choline) is present in this ingredient, as it may have happened with meat scraps or fish based products.

Studies carried out in the 1970’s [75] demonstrated that *T. molitor* larvae require 10 essential amino acids, which are quite similar to human needs. Based on this information, blood might be the most preferable source of essential amino acids, since practically 60% of its amino acid composition accounts for essential amino acids. On the contrary, offal rich in collagen will only provide around a 25% or 17% of essential amino acids, for example, lips or ear, respectively. Other common co-products such as brain, kidney, liver or lung range from 40% to 48% content in essential amino acids [72,73].

Regarding vitamins, it has been reported that vitamins B1, B2, B3 and B5 are essential or beneficial for insects [76]. This is of special interest when formulating insect substrate including meat co-products since most of them are excellent source of vitamins from the B group, being kidney and liver the richest sources among them [72,73].

When considering minerals, those considered essential are calcium, chlorine, copper, iron, magnesium, manganese, phosphorus, potassium, sodium, sulphur and zinc [77]. Based on the mineral profile of meat co-products, it can be stated that all these minerals are present in diverse amounts. In particular, offal is a rich source of iron and zinc [72,73].

Finally, some lipids are regarded as essential in insects’ diet, for example, cholesterol, linoleic acid and linolenic acid [77]. Some of the offal feasible to be used in substrate formulations are a source of both linoleic and linolenic acids; for instance, liver and kidney have been reported to have 0.04–0.05 and 0.01–0.10 g of linolenic acid per 100 g, while linoleic acid was found to be 0.15–0.19 and 0.15–0.47 g/100 g relative to the kidney and liver, respectively. The heart from pork is also a source of these nutrients [72,73].

Based on this information, there is a potential for including animal co-products as substrate for insect rearing in order to supply the required essential amino acids, minerals and vitamins, while collagen can not only be used as a source of proteins but also as functional ingredient to provide texture and consistency to the substrate. However, there is a lack of research regarding the impact of using meat co-products as substrate for rearing insects, and the impact on production parameters such as survival, weight, size and life cycle needs to be investigated.

## 3. Valorisation of Side Streams in Insect Feed

As discussed above, insects are able to efficiently convert low-value biomass into high-value substances and, therefore, might have the potential to valorise organic side streams. However, integrated valorisation of biomass often faces some challenges. The most important challenge is to collect the biomass. The (local) available biomass is often limited, and there is a lack of continuous supply (time and volume) of the biomass. Moreover, collection, transport and processing of biomass can result in high production costs. Other challenges may include legislation and also the very important perception of the society [78].

An important hurdle of using organic side streams are the legal aspects. According to the EU Regulation 2017/893, insects are considered farmed animal; therefore, rules for animal feeding must be observed following EC 1069/2009. Hence, insects can only be produced with substrates eligible as feed materials for farmed animals. Thus, it is prohibited to use certain materials such as slaughterhouse or rendering-derived products, manure, catering waste or unsold products from food industries or retailers that contain meat or fish. The regulation on animal feed marketing allows insects to be fed with materials of vegetal origin if they are not defined as waste, as well as some materials of animal origin such as milk, eggs, honey, rendered fat and blood products from non-ruminants [79].

For efficient valorisation, it is important that the side streams are used for an application as high as possible in value. The hierarchy of values for biomass applications is drawn up as follows: (1) food for human consumption, (2) feed for animals, (3) functional materials and products and (4) fuels and their applications [80]. Moreover, it is also important to mention the fact that valorising residues into animal feed is not a form of waste treatment, as is the case for bioenergy and compost applications [81]. Therefore, valorising side streams as animal feed is preferred by many sectors instead of sending it to waste processing and may generate a financial advantage. When using side streams, it is best to check its current application(s), and it important to avoid competition with other sectors, as the availability of the biomass may depend on this. According to the European Former Foodstuff Processors Association (EFFPA), an estimated 5 million tons of former food stuff is currently used as animal feed in the EU. They also claim that this can increase to 7 million tons until 2025 [7]. Moreover, it has been reported that the most promising approach for the insect production industry includes the use of not yet or low valorised agricultural products [82]. Therefore, focus must be laid on the investigation of low valorised biomass, i.e., used for energy production, land filling or discard or oversupplied biomass such as potential insect feed.

## 4. Dietary Requirements of the Insects in Scope

In order to explore the valorisation potential of side streams such as insect feed, it must be considered that the biomass complies with the nutritional and physical requirements insects place on their diet. The dietary requirements for *T. molitor*, *A. domesticus* and *L. migratoria* are discussed below.

### 4.1. Tenebrio molitor

When rearing mealworms, a distinction is often made between the dry feed source in which the insects thrive and the wet feed source that is added for provision of moisture. Liu et al. [83] reported that supplementation of wheat bran with approximately 30% fresh plant materials significantly affects mealworm growth rate and final weight (40–46% heavier). Mealworm larvae were grown on wheat bran or wheat bran enriched with carrot, orange or red cabbage. Oonincx et al. [12] also reported that supplementing with a wet feed source (e.g., carrot) reduces development time and mortality.

*T. molitor* is generally fed with a dry feed such as bran [84]. Depending on the amino acid compositions, the protein concentration can be as low as 10% [85], but in general they prefer a higher concentration (28%) [43]. The study of Davis [75] showed that ten essential amino acids (histidine, lysine, arginine, isoleucine, tryptophan, methionine, leucine, phenylalanine, valine and threonine) are required in the diet of *T. molitor*. A deficit of these amino acids will adversely affect the growth of *T. molitor*; however, the survival rate was not affected by the deficit of an amino acid.

The carbohydrate concentration should be high, 50% or higher and can consist entirely of starch. In all cases the diet should include vitamins of the B-complex [84]. A good standard diet can be as follows: 80% wheat bran and 20% supplement consisting of 83% dry potato, 13% dry egg white, 2% soy protein and 2% peanut oil [43].

### 4.2. Acheta domesticus

Patton [56], reported four diets that produced satisfactory results based on the survival and growth rate of *A. domesticus*. The protein content of these diets varied between 20% and 30% (on dry matter basis), carbohydrates from 32% to 47% and fat ranging between 3.2% and 5.2%. However, they used several ingredients of animal origin. Morales-Ramos et al. [86], designed several cricket diets through a self-selection process, concluding that vitamin B and C, sterols and manganese had significant positive impact on live biomass production.

### 4.3. Locusta migratoria

Different attempts were made to construct artificial diets for *L. migratoria*, particularly by Dadd in the early 1960s [62,63,64,65,66]. However, since *L. migratoria* is a specialist, none were as successful as their natural food sources, i.e., grassy biomass. As long as fresh grass is provided daily to the locusts, they do not need to be provided with an additional moisture source (e.g., water) [87,88,89]. Through his research, Dadd gained a lot of insights regarding the nutritional needs of *L. migratoria*. For the nymphs to grow sufficiently, the diet had to contain 20% protein (DM basis), 10% of digestible carbohydrates, linoleic acid (0.5%), cholesterol (0.5%), ascorbic acid (0.3%) and vitamins (0.2%). Moreover, cellulose seemed to be required (66.1%) despite it is indigestible for locusts [62,63,64,65,66]. The specialist grass-feeder *L. migratoria* was evaluated in its diet selection and response to macronutrient imbalances. In ad libitum conditions, *L. migratoria* composed a balanced intake of proteins and carbohydrates, and they regulated ingestion when confined to imbalanced foods in such a way as to mitigate excesses as well as deficits of macronutrients [60].

In general, the minerals calcium, chloride, copper, iron, magnesium, manganese, phosphorus, potassium, sodium and zinc are vital in small quantities relative to many insect species [77]. These minerals are, therefore, considered essential in this review; however, to the best of our knowledge, no specific information regarding minerals for the insect species in scope could be found. Regarding vitamins, those from the B-complex are essential for all insect taxa or at least found beneficial for obtaining normal growth and development in captivity. Meanwhile, vitamins other than vitamin C, B8, B9 or choline are only essential for specific taxa [76].

It has been previously reported that mono streams do not seem to meet the nutritional needs of the insect species in question. Moreover, diets composed mainly of organic waste streams or by-products may cause lower growth performance and survival of insects. The insects might also behave cannibalistically when the diets given are lacking in nutrients, with an even higher mortality as a result. The given diet must be supplemented with high-quality products in order to ensure proper development of the insect [54,57,67]. An advantage of mealworms and crickets is that they thrive on a dry substrate supplemented with a source of moisture. As long as the dry feed is nutritionally balanced, the nutritional value of the moisture source is irrelevant [90]. This opens up more possibilities to use moist side streams as wet feed for insects.

## 5. Influence of Diet on Nutritional Value of Insects

Insects are reported to be highly nutritious and to be a good source of proteins and fats. A thorough overview on the nutritional composition of more than 200 edible insects has been published by Rumpold and Schlüter [25], and it indicates that many insects provide satisfactory levels of proteins, energy, MUFA’s and PUFAs, as well as several micronutrients and vitamins, making them potentially suitable for food, feed and technical applications. However, when considering insects for such applications, the reflection of the feed given to the insects must be understood, especially when feeding side streams, as the diet influences the chemical composition and nutritional quality and, thus, may also influence the acceptability of insects [91].

It also needs to be noted that, apart from the influence of substrates, other factors including killing (e.g., freezing or blanching) and processing (freeze drying and oven drying, etc.) of the insects may also impact their nutritional value, as well as processing steps (e.g., chemical extractions) that are needed to implement insects or insect-derived products (e.g., proteins or fats) in different types of applications [91,92,93,94].

The discussion below is limited to an overview of the effect of organic side streams that are used as substrate on the protein and fat fractions of *T. molitor*, *A. domesticus* and *L. migratoria*. For information on the composition of the diets used in the discussed studies, the reader is referred to Table 1 and Table 2.

### 5.1. Tenebrio molitor

The observed large variability in lipid (range 6–40%) and protein (range 36–76%) levels in mealworms among different studies [11,12,26,27,40,44,45] may reflect an influence of the substrate and/or rearing conditions on the proximate composition of the mealworms. It has been suggested by several authors that the nutritional composition of insects can be altered by diet; however, the underlying mechanisms that steer this nutritional composition are little understood.

Ruschioni et al. [45], showed the potential of mealworms to convert low-value organic by-products (olive pomace) into higher-value biomass. Despite relatively low protein content of the substrates (ranging from 11.76% to 15.42% DM), mealworms were obtained with up to 47.58% DM protein content. Mealworm larvae mirrored the proximate data of their substrates, and protein content and essential/nonessential amino acid ratio were different depending on the substrate. The proximate composition of the substrate is not always reflected in the larvae. For example, Zhang et al. [26], studied the potential of three agri-food industry by-products (mushroom spent corn stover (MSCS; 3.9% DW protein DW, 12.8% DW lipids), highly denatured soybean meal (HDSM; 43.2% DW protein, 4.8% DW lipids) and spirit distillers’ grains (SDG; 13.9% DW protein, 6.9% DW lipids) as feed for *T. molitor*. Even though the MSCS and SDG treatments had lower protein content compared to control (22.5% DW protein, 7.3% DW lipids), protein and fat fractions ranged from 70 to 76% and 6 to 12% of mealworm dry weight on the three treatments, respectively. All mealworm larvae had higher protein and carbohydrate content compared to larvae bred on wheat bran regardless of the amount of proteins in the substrates. This indicates that nutritionally-poor substrates can still yield nutritionally interesting mealworm larvae. However, it must be noted, for possible food applications, that several indispensable amino acids for some diets were lower than the FAO requirements (leucine, lysine, methionine + cysteine and valine). Furthermore, diets are observed to affect the amino acid composition in the larvae, but there is no pattern in how the levels in the diet affect the levels in the larvae. Zhang et al. [26] suggested that protein denaturation may play a role. In the study of Melis et al. [44], mealworm larvae reared on Brewer’s Spent Grains (BSG) showed slightly higher crude protein (14.78% on fresh weight basis (FW)), but conversely a significantly lower amount of crude fat (6.39% FW) compared to larvae fed with the control diet (13.35% and 12.42% on FW, respectively), even though the BSG diet contained higher crude fat levels than the control diet (7.30% versus 4.08%). In this study, fat content of the diet was not reflected in the larvae, but protein content was since the BSG diet showed slightly higher protein content (22.45% on DW) than the control diet (19.57% on DW). Harsányi et al. [40] reported that mealworm larvae showed limited variation in nutritional composition despite substantial variation in nutritional composition of the substrates on which they were raised (substrates containing each 90% of vegetable waste, garden waste, cattle manure and horse manure). Protein content in the larvae ranged from 37.9% DW to 47.18% DW (substrates ranged from 2.8–21.3% DW protein) and lipids ranged from 43.08% DW to 47.5% DW (substrates ranged from 2.34% DW–5.58% DW lipid). These results also indicate that mealworms are able to convert low value streams into nutrient-rich biomass. In the study of Oonincx et al. [12] in which mealworm larvae were bred on four diets defined by varying protein and fat content (mixtures of spent grains, beer yeast and cookie remains), it was shown that larvae that were reared on high protein diets also had higher protein levels; however, the effect of lipids in the substrates was not clearly reflected in the larvae. Protein contents of the larvae ranged between 44.1% and 53.6%, while the protein contents of the diets ranged between 12.9% and 22.9%. In contrast, van Broekhoven et al. [11], who tested four mixtures of maize DDGS, beer yeast, bread remains and potato peelings as mealworm feed, indicated that the protein fraction in larvae was similar (46.9–48.6%) despite 2-fold to 3-fold differences in protein content in the substrate (range 11.9–39.1%). The authors suggested that mealworms are able to regulate body protein content. A slight correlation was observed for fat content in substrate and larvae. Moreover, Li et al. [27] observed that protein and fat contents of mealworm larvae reared on fermented plant wastes reached 76.14% ± 0.74 and 6.44% ± 0.56 on dry weight basis, while the protein and fat contents of larvae reared on the control diet were 68.15% ± 0.66 and 17.43% ± 0.05, confirming the potential to rear nutritional-rich mealworm larvae on low-value side streams.

### 5.2. Acheta domesticus

Nutritional data of house crickets also seem to have a large variability in lipid (range 6–21%) and protein (range 56–67%) levels among different studies [12,40,54,95], probably due to the different diets used in these studies. Most studies indicate that crickets are very rich in proteins and less in fats, and their diet can affect the nutritional composition to some limited extent. In the study of Oonincx et al. [12], house crickets were fed with four diets defined by a varying protein and fat content and composed by mixtures of spent grains, beer yeast and cookie remains. In their study, survival rate was low compared to crickets fed on control diet. Food conversion ratio and nitrogen efficiency were also suboptimal, possibly due to insufficient water provision and/or the presence of a densovirus in the facility. Only crickets fed on the high protein diet and on the control diet were analysed and had a high crude protein content (58–59%) and a low lipid (17–21%) composition. The high variation in fat composition of the diets is not reflected in the composition of the crickets. The main fatty acid was C18:2 n6, although C16:0 and C18:1 n9 were also present in high concentrations. Large differences in C18:2 n6 and α-linolenic acid (C18:3 n3) concentrations were found due to dietary treatment. The n6/n3 ratio ranged from 15.3–29. C20:3 n3 and C22:6 n3 were not detected in the diet but were present in the house crickets; this could suggest de novo synthesis of these fatty acids. Collavo et al. [54], exposed crickets to diverse diets and evaluated yield and survival. Unfortunately, the nutritional composition of the substrates was not reported. The protein content of crickets ranged from 56.2% up to 60%, and the fat content ranged from 6.3% to 12.2%. The amino acid distribution was similar among crickets that were fed the different diets, and the quality of the proteins was good. The fatty acid profile of the insects mainly contained palmitic acid, oleic acid and the essential fatty acids linoleic acid and α-linolenic acid. Harsányi et al. [40] also investigated the effect of diet on house cricket composition, rearing them on mixtures (90% waste, 10% chicken feed) containing vegetable waste, garden waste, cattle manure and horse manure. They observed that house crickets are specifically high in protein (56.4–67%) and less in fats (14.41–19.4%). Feeding crickets with nutrient-poor diets resulted in crickets with the lowest protein and highest fat content; however, the nutritional composition of the crickets is only affected to a limited extent by the diet.

Bawa et al. [95], evaluated the growth and composition of crickets reared on five diets differing in crude protein (6.3–21.9% DW), crude fat (1.4–5.2% DW), crude fibre, crude ash and carbohydrate (65–79.4% DW) content. Crickets reared on protein rich diets performed slightly better, but there were no differences in survival rates. However, the proximate composition in the crickets was significantly influenced by the diet. Significant differences were observed in the quantity of crude protein (range 48.06–76.19% DW), crude fat (range 8.9–43.9% DW), dry matter, crude fiber, crude ash and carbohydrate (range 5.1–10.3% DW) content. Crickets fed on a high protein content diet had high protein and low fat content. Adding dry pulp pumpkin powder to the diet improved the vitamin B content.

### 5.3. Locusta migratoria

To the best of our knowledge, no studies on the production of *L. migratoria* on side streams have been reported. However, it has been reported that the composition of *L. migratoria* can be altered by diet. In the study of Oonincx and Van Der Poel [96], migratory locusts were fed with a diet containing fresh perennial ryegrass (FPR) or FPR + wheat bran or FPR + wheat bran + carrots. Adding wheat bran decreased the protein content from 63.3% to 58.3% and increased fat content. Additionally, adding carrots to the diet further decreased protein content and increased lipid content from 18.2% to 23.1%. These observations were conducted for both the juvenile as the adult locusts. Mineral concentrations of Ca, K, Mg and Na were significantly affected by diet. Concentrations of Ca, K and Na decreased when wheat bran was provided. Wheat bran decreased the α-carotene content, which did not change by incorporating carrots in the diet. However, carrots did result in higher β-carotene concentrations. Retinol concentrations were increased by incorporating both wheat bran and carrots in the diet compared to the diet containing only grass.

## 6. Agro-Food Side Streams Availability in the European Union

Along with biomass production and consumption, biodegradable side streams and residues are generated. This review focusses on biomass side streams and residues created along the agro-food supply chain at European Level.

In order to be able to categorise the side streams, a distinction is made between the various sectors of the agro-food supply chain. In every sector of this agro-food supply, chain losses are produced. In the past few years, several studies have attempted to provide a quantitative overview of the losses that are created along the food supply chain [6,97,98]. Current estimations of food loss and waste generation in Europe ranges between 158 and 298 kg per person per year [97].

In this section, relevant examples of side streams and residues at the level of primary production, processing and manufacturing, wholesale and retail are discussed. Although a substantial share of waste is created at the level of food services and households [5,98], these sectors will not be discussed here due to legal aspects, as described above. Since these streams cannot be used as animal feed [79], they were found irrelevant for purposes of this review.

The availability of waste streams also influences the actual valorisation potential in insect feed, as the supply will depend on this. Therefore, side streams that are highly available and are currently low valorised are explored as potential insect substrate. Current applications of side streams are discussed.

Numerous studies have been conducted attempting to estimate the availability of agro-food residues at EU-level. However, it must be noted that, despite a series of shortcomings, these data are clearly identifiable. In fact, hard data are often not collected at the EU-level, and estimations are often based on assumptions.

### 6.1. At Primary Production Level

Focus is laid on agricultural crops since animal by-products (except for manure) are mainly produced during slaughter, i.e., processing (see further).

Agricultural crop residues can be defined as the part of the plant that is left over after harvest. The residues vary greatly per crop in shape, structure, composition and decomposition rate [99]. They mainly consist of foliage and stalks of plants and cannot be consumed as food [100]. The total estimated production of agricultural crops in the EU(28) was 649 million tons in 2018; this corresponds with an estimated production of 626 million tons crop residues [101,102].

In the table below (Table 3), data from FAOSTAT [102] on the agricultural crop production are presented, as well as the estimated availability (selected on highest EU production quantities) of crop residues (total crop residues, not corrected for valorised applications). For the sake of completeness, the totals per commodity are also given. Estimated quantities of crop residues are calculated using the ratios calculated by Ronzon et al. [101], based on the study of Wirsenius [103]. These ratios were calculated by dividing the unconsumable part of the plant by the consumable part of the plant, which is the product, and its quantity can be found in the FAO tables. Data are presented at the EU(28)-level.

Although some agricultural side streams are already being used for animal feed applications, their total use in animal nutrition is still limited regarding the production amounts. For instance, only 3.3% of the total food surplus is processed into animal feed in the EU (EFFPA, 2018) [7,98,104]. Moreover, according to the study of Caldeira et al. [98], 9.46% of the agricultural crop production from the FAOSTAT database (2011) is used for non-food purposes (low valorised), and 4.62% of the total crop production is wasted (25% of total food waste in the food supply chain) (calculated on data of the year 2011). This indicates that mostly residues from primary production have valorisation potential from all sectors in the food supply chain (if we exclude the household sector). Around 45% of the amount fruit and vegetables waste entering the food supply chain is wasted at the primary production level. This is due to the high share of inedible parts in these products and also to the higher perishability of fruit and vegetables compared to for example cereal-based products (e.g., flour and rice) [98]. Furthermore, cellulose-rich agricultural crop residues (e.g., foliage) are often reused for low valorised agricultural applications, such as animal bedding or for the production of renewable energy. However, a substantial amount of agricultural residues is currently not used for any productive purpose. It is placed into landfills, incinerated or other handled with other disposal methods [100]. In the study of Searle [105], the total potential of agricultural crop residues (barley, corn, oats, olives, rapeseed, rice, rye, soybeans, sunflower, triticale, wheat and sugar beet) in the EU(28) was estimated for energy applications. It was estimated that 62% of the agricultural crop residues need to be left on the field since they cannot be feasibly harvested. This includes the minimal quantities of residues to be left on the fields for sustainable agriculture (soil protection) and is, therefore, considered unusable for valorisation in this review. Taking this into account, at least 30% of agricultural crop residues possess valorisation potential [105]. This unusable part is not included in the RTP ratios calculated by Ronzon [101] and Wirsenius [103].

The losses in primary production for fruit and vegetables estimated by Caldeira [98] correspond to the residues calculated by the ratios of Ronzon. However, this is not the case for cereals because Ronzon’s RTP ratio is much higher than the coefficient used by Caldeira. Caldeira only includes the part of the plant in its coefficients that can be used for animal and human consumption, while Ronzon looks at the whole plant and also includes the non-consumable parts, such as straw.

### 6.2. At Processing and Manufacturing Level

When food is processed, large amounts of side streams are generated. It is estimated that between 16.9 and 30.6 Mt of side streams generated at this stage of the food supply chain are not valorized and disposed of to a waste processing facility [6,98]. These estimates include both (edible) food and inedible parts associated with food.

It is important to note that a lot of the side streams generated during processing are already recovered as animal feed or non-food applications. In fact, according to Caldeira et al. [98], 60% of the side streams are already valorised in animal feed, and 20% of side streams are used in non-food applications, leaving only 24% of non-valorised side streams. For example, during the processing of sugar beet from the 6 Mt of residue, 4–5 Mt goes to animal feed, according to Kemna and van Holsteijn [106].

The highest share of non-valorized side streams generated at processing and manufacturing level are oil crops (32%), followed by fruits and vegetables (19.9% and 8.4%) and fish (10.1%) and meat (9.4%) [98].

Valorization of fruit and vegetable side streams is challenging owing to their perishable nature and heterogeneity, among other factors.

According to the study of Caldeira et al. [98], 6.1 Mt (million tons) of fruit and 2.6 Mt of vegetables become lost at processing and manufacturing level in the EU, which accounts for 5% (fruit) and 2% (vegetables) of the total food waste (non-valorised). It was also reported by FAO [5] that an estimated 2% of fruits and vegetables becomes lost at the processing and packaging level in Europe.

Animal by-products such as fish and meat contribute to an estimated 2.4% and 2.2% of the total food waste produced at processing and manufacturing level. Fish by-products actually contribute to a large part to the wastes produced at processing and manufacturing level, considering that these products are consumed in smaller quantities than for example meat or fruit and vegetables [98]. By-products from the fish processing industry are not usually valorised (except for fish meal and oil) [107] contrary to what happens, for example, with meat. Meat by-products also generate a large amount of inedible parts (e.g., bones, skin, wool and feathers), where a high share of them is being used in other industries [108] (see further).

Based on the information gathered from FAOSTAT [102], the number of porcine and cattle heads that have been slaughtered has varied in opposite directions within the European territory. While pork heads have increased with 1.89% from 2010 to 2018, in the same period of time, the cattle heads have decreased with 7.48% from 2010 to 2018. Poultry production has been continuously growing over the past decade, which indicates there is an increasing production of poultry by-products, such as blood and feathers.

To provide a detailed look at the situation in the EU(28), the number of heads slaughtered is presented in Table 4.

The data indicate that the overall number of porcine and poultry heads has increased over time, although not significantly. The production of offal and co-products is expected to increase in a similar percentage.

Based on previous reports published by the Southampton University and Ashtown Teagasc [109], the percentage of the animal live weight considered as offal, co-products or edible by-products can be as high as 25–30% for cattle (excluding fat or Category 1 and 2 products) and around 20% for pork (excluding fat and Category 2 products). Feathers can be up to the 10% of the body weight of the chicken, although regular values range from 4% to 6%. This means that considering an average bodyweight of 1.5 kg for poultry, 100 kg for pork and 550 kg per cattle, the amount of non-meat products that are generated annually is quite large. As presented in Table 5, the annual production in 2018 was 9,294,053 tons of offal and co-products and around 546,410 tons of feathers in the EU(28). Out of this, the vast majority was used for rendering, land filling, pet food and, in a minor percentage, for food [110].

Mostly due to restrictions imposed by legislation, the valorisation of meat co-products in animal feed is quite limited with the remarkable exception of plasma from pork blood, which is used for broilers, piglets and dairy cows feeding and, more recently, for aquaculture purposes [111,112]. However, the red cells fraction is still not exploited for these purposes. Other co-products, such as tongue, lungs, heart, kidneys or spleen, have a limited market for human consumption, which is decreasing continuously and is mostly employed as ingredient for pet food and very specific industrial applications (see Table 6), although not at great extent since the most of them are used for land filling or composting [72].

### 6.3. At Wholesale and Retail Level

The auction system that typically governs wholesale markets generates losses. Buyers bid on both the quality and quantity of items, resulting in unsold products. Moreover, wholesale markets clear their inventory of perishable products at the end of the business day to ensure that there is adequate space for the following day’s auction items [113]. Major players in the wholesale and retail sector include the fruit and vegetable auctions, which receive and sell fresh fruit and vegetables. The products sold vary within organizations and according to the season. Unfortunately, many products do not end up on auction or are not sold due to overproduction. Other reasons for discarding crops include deviating colours, shapes or sizes that do not comply with the auction’s criteria. Moreover, damaged crops are also discarded [113,114]. According to Stenmarck et al. [6] and Caldeira et al. [98], approximately 5% of the total amount of food waste in the EU (non-valorised) is generated in the wholesale and retail sector. The largest contributors to the waste at the wholesale and retail level are cereals (25% of the sector, 1.3% of total food waste), meat (25% of the sector, 1.3% of total food waste) and fruit and vegetables (12% and 13% of the sector; 0.6% and 0.7% of total food waste) [98].

Most of the residues created at wholesale and retail level are valorised in animal feed or redistribution (e.g., food banks). Other destinations include fermentation, land filling and composting [115]. To the best of our knowledge, no estimations on low valorised wholesale and retail products at EU-level were made; however, according to Belgian data almost half of the residues at retail level in Flanders are anaerobically digested [115]. Regarding fruit and vegetables auctions, according to Belgian data approximately 43% of the fruit and vegetable losses from auction have low valorised applications (fermenting and land filling) [116].

## 7. Discussion

The aim of this review is to provide more insight on the use of organic side streams as potential feed for *T. molitor*, *A. domesticus* and *L. migratoria*. The valorisation potential of the use of agricultural side streams for the rearing of the insects in scope was explored. The different points to be taken into account when valorising side streams in insect feed were discussed.

### 7.1. Legal Aspects

First of all, when aiming to valorise side streams in insect feed, the legal aspects are a very important factor to consider. Not all organic biomass can be used as feed for insects according to the EU legislation. Insects are considered farmed animals and, therefore, must follow legislation of current livestock [79]. Although household waste contributes the most to food waste [5,98], this stream currently cannot be used as feed. The same accounts for other streams, such as manure, slaughterhouse or rendering-derived products and catering waste. However, some streams are allowed to be used, for example, non-waste materials of vegetal origin, milk, eggs, honey, rendered fat and blood products from non-ruminants [79].

### 7.2. Side Stream Value and Availability

Furthermore, it is important to investigate whether the biomass is not yet valorised in animal feed. Currently, insect production is a relatively new concept in the EU, making it difficult for insect breeders to compete with conventional livestock farmers for feed. Additionally, the availability of side streams depends on the current applications. Therefore, focus must be laid on low valorised side streams, e.g., biomass used for energy production, land filling or disposal. Moreover, biomass produced in oversupply has potential as insect feed, even though it might already be valorised in minimal amounts. Moreover, it has been reported that the most promising approach regarding profitability for the insect production industry includes the use of not yet or low valorised agricultural products [82].

Thus, the availability of low valorised side streams must be considered. Not all biomass is available in large quantities nor during a year-round period [117]. This makes it challenging to implement biomass in insect feed, since the feed is preferred to be standardized in order to avoid fluctuations in, e.g., yield or nutritional composition of the insects produced [28]. This also includes seasonal and country related differences in the supply of the biomass. For example, some food products and, therefore, side streams are primary produced in specific countries due to factors such as climate. Furthermore, it must be considered that fresh materials might not be easy to transport to other regions, especially low-density materials that increase the costs of collection, handling, transport and storage [117,118,119]. Such examples include streams to be used as insect wet feed (*T. molitor* and *A. domesticus*), especially for *L. migratoria* which primary feeds on fresh grass [58,59]. Streams might be further processed to become storable products (e.g., drying, freeze-drying and fermentation) as this has been successfully tested [120,121]; however, further processing also increases production costs [82].

### 7.3. Side Streams Produced at Different Levels

This review explored the availability of agricultural residues produced at the EU-level; however, the EU covers a large area, and it is recommended to consider local supplies of side streams due to above-described reasons.

Side streams are produced within all sectors of the food supply chain. Looking at the data, most residues are produced at the primary production level. This makes sense since this is the first sector of the food supply chain. Moreover, during primary production, harvesting takes place and some products such as fruit and vegetables contain a high fraction of inedible parts, i.e., residues [98]. However, not all residues at primary production level can be used in insect feed. Some products such as cereal residues are already valorised in animal feed [98,106,122]. Moreover, marketable products are often valorised in food and feed products. In addition, fresh products such as fruit and vegetables have high perishability, making it more difficult to transport or store these products and increasing production costs [115,117,118,119]. Therefore, most of the inedible parts of primary production products must be considered for valorisation. These residues are currently used for low value applications such as animal bedding and energy production or are even discarded [100]. However, it must be considered that these products often are available in raw form and, therefore, might need to undergo pretreatments to render them suitable as insect feed, increasing the production costs. Furthermore, a large part (an estimated 62%) of the residues is considered unusable for valorisation since it contributes to the soils health and, therefore, needs to be left on the field [105].

Since a lot of residues at primary production level are not yet valorised or used for low valorised applications [98], there still is a valorisation potential. It can be concluded that residues with the most valorisation potential include horticultural foliage such as from tomatoes, bell pepper and strawberries, etc. However, it must be taken into account that these products might be seasonally produced. Fresh products can be valorised if they can be found locally with high availability and can be stored (e.g., pretreated) or are produced year-round.

Side streams created at the processing and manufacturing level often contain high amounts of nutrients and might need less pretreatment. Currently, a lot of these side streams are already valorised in animal feed. However, low valorised by-products and wastes are still created and can be considered for valorisation. According to Caldeira et al. [98] an estimated 30.6 Mt by-products produced at processing are wasted in the EU. Side streams with the most potential according to availability include fruit and vegetable by-products and animal by-products from meat and fish [98,123]. For example, the red blood cell fraction (from non-ruminant blood, referring to the EU legislation [79]) is not yet exploited for livestock feed.

At the wholesale and retail level, the food loss is smaller compared to the other stages of the food supply chain [98,113]. Therefore, the availability of products to potentially be valorised is lower. However, we must mention that data on the availability of products in this sector are still missing. Additionally, since the side streams mainly consist of marketable products, those products are often valorised by redistribution (donation) and in animal feed. The largest contributors to side streams in this sector are cereals, meat and fruit and vegetables (e.g., from the fresh market/auctions) [98,115]. Since the residues created at wholesale and retail level are small (5% of total EU food waste) [6,98], it must be taken into account that these products can only be considered as insect feed when a continuous local supply is available.

### 7.4. Insect Diet Requirements

As discussed in this review, several studies have already been focusing on rearing insects on organic by-products. It can be concluded that there is definitely potential for using side streams as insect feed, as long as their dietary requirements are respected. Although insects are considered to be able to convert low-value biomass, they also have certain demands regarding their diet, both physically and nutritionally. The current issue with this is that knowledge is still lacking. Detailed information on certain components and per insect species (especially for house crickets and migratory locusts) is still missing.

This review has shown that most insects cannot be grown on mono streams only, as these streams often have an unsuitable moisture content, texture or do not meet the nutritional needs of the insects [46,54,57,67]. Therefore, compound diets that are a mixture of side streams for increasing the nutritional value might be more suitable, taking into account the physical diet requirements of the insect species in scope. As these requirements are very species dependent, it might be challenging to compose diets for some species, for example, house crickets, migratory locusts and especially mealworms who reside in their substrate. Mealworms, for example, thrive on a dry feed, making it more difficult to include moist biomass or compound suitable diets without using a lot of processing or pretreatment steps, while for some insects (e.g., black soldier fly) moist mixtures are much more accepted by the insects as feed [124,125]. Furthermore, it has been suggested that other species, such as the black soldier fly, might be better suited for converting low-quality organic side-streams to high-value components, as they can thrive on many waste streams [57,125]. On the other hand, species related dietary requirements might create possibilities for biomass valorisation. It was proven by several studies that mealworms and crickets have potential to be fed with organic side streams, as discussed in this review. Furthermore, the insects in scope still receive some moisture of their feed. It has been proven that supplementing the dry feed with a moisture source, so called wet feed, reduces mortality and development time of mealworms [12]. Moreover, as the only purpose of this wet feed is to provide the insects with moisture [90], this can be a mono stream, which increases the potential use of vegetal residues for this purpose.

### 7.5. Additional Issues and Opportunities

Another factor to be considered is the nutritional value of the produced insects on organic biomass. Through this review, it can be concluded that insects can be reared on biomass low in nutrients and still contain high amounts of valuable nutrients such as protein and fat. For example, in the study of Zhang et al. [26], mealworms containing up to 76% protein were obtained using agri-food industry by-products as insect feed.

The desired composition of the insects often depends on the application of the end product. As discussed, it is suggested by several studies that the nutritional value of the insects can be manipulated by diet; however, this is currently only marginally understood.

Other factors, including genetics as well as variations in the intestinal microbial populations within insects, may influence the bioconversion efficiency of diets by insects, as well as influence their nutritional composition [126,127,128]. Selective breeding is expected to play an important role for the bioconversion of specific residues by insects. Since insects have short lifespans and high reproductive rates, insect adaptation (evolution) may occur within economically relevant time scales [129].

Recent work on the black soldier fly, using 15 microsatellite genetic markers, captured the genetic diversity of global and domesticated BSF populations and suggests the importance of the genetic make-up of BSF populations [130]. The recent sequencing of the BSF genome and the genetic manipulation of BSF towards an enhanced feeding capacity phenotype [126] further illustrate future opportunities for more efficient insect farming. More research studies must be conducted in order to be able to steer the nutritional value of the insects. If this is possible, mealworms and crickets could be reared with a specific composition, which would lend itself for specific applications. Rearing those insects on agricultural residues would lower production costs, which is beneficial to those applications and the sector. It can be concluded that much research still needs to be conducted in order to be able to map the full potential of organic biomass as insects feed. After all, the industrial rearing of insects is still in an initial phase. However, bioconversion of side streams by insects has potential in future, with growth in the insect business [125,129]. The challenge here is to produce the insects optimally, i.e., profitable (e.g., low FCR, high yield) and sustainable, by using organic biomass often lacking in required nutrients. Regarding the challenges the society is facing today, focus must be laid on sustainable production of the insects, referring to limiting pretreatment or processing of organic biomass.

It must also be considered that organic wastes rich in nutrient and water content are prone to faster dissolution. This might result in odour problems and potential manifestation of microorganisms. Pretreatment (e.g., fermentation) of the organic wastes can be conducted for the waste stabilization. Moreover, most nutrients present in agricultural residue or by-products are in insoluble form and fermentation can help in enhancing the digestibility and bioavailability of nutrients to the insects [28,131].

Although it might be challenging to valorise side streams in insect production as different points must be considered, it is not impossible. After all, a lot of organic waste is produced at EU-level. In fact, insect-based bioconversion represents a promising economic sense for businesses.

Today, few companies in the EU already address the issue of organic waste by insect-based bioconversion. For example, Innovafeed uses wheat by-products as feed. Moreover, other companies such as Ynsect, Protifarm, Protix, Goterra, NextAlim, nextProtein and Entofood are already operational in the use of a variety of organic biomass for insect production. This indicates that the use of organic residues for the industrial mass production of insects such as *T. molitor*, *A. domesticus* and *L. migratoria* still needs to be finetuned; however, this industry does have a huge potential.

Although not discussed in this review, food safety must be considered when using insects for certain applications (e.g., food and feed), especially when they are produced on side streams. The insects’ feed might introduce safety risks by microbial contamination and accumulation of several compounds in the insects, such as toxic metals, mycotoxins and pesticides [132,133], etc. Although research has been conducted regarding this topic, knowledge gaps are still present, which is mainly the reason why the European Union currently does not allow the use of several side streams as insects substrate. Further research must be conducted in order to ensure safe production of insects on side streams.

## 8. Conclusions

The aim of this review was to explore the valorisation potential of agricultural side streams as feed for the rearing of yellow mealworm (*T. molitor*), house cricket (*A. domesticus*) and migratory locust (*L. migratoria*) under a European perspective. While collecting the relevant data for this review, it was observed that there are many inconsistencies in literature regarding the calculation of FCR (fresh weight, dry weight bases and combinations thereof) and other parameters. An international working group has been established during the EAAP 2019 conference, aiming at the standardization of methods, parameters and terminology in insect research [134]. This initiative will be very helpful for comparing future studies in insect research. A recent paper by Bosch et al. focuses on the possible sources of the variability observed among BSF feeding experiments and argues for the development of procedures in order to improve harmonization and reproducibility among studies [135].

Through this review, it can be concluded that there is huge potential to valorise residues in feed for the insects in scope. Many organic residues are produced at EU-level, and studies investigating insect-based bioconversion report promising results.

However, valorising side streams in insect feed might be challenging since several aspects must be considered regarding European legislation, the insects’ dietary requirements, the nutritional composition of insects and the local availability of agricultural side streams. Not all side streams from the food supply chain can be used, since the use of some streams is limited either due to legal aspects, seasonality, those that are already valorised, transport and storability of fresh streams or unsuitability for insect production.

It can be concluded that side streams are produced within the entire food supply chain; however, streams with the highest availability are mostly produced at the beginning of the chain, i.e., primary production, processing and manufacturing level. Streams with lower availability can still be valorised in insect feed if the streams are locally available.

Taking the above-described considerations into account, streams with the highest valorisation potential include those that are not yet valorised or low valorised. Potential insect feed should not compete with current applications such as animal feed, since the availability of the streams will depend on this. Furthermore, valorising low-value streams in insect feed would lower insect production costs. In addition, local streams, especially when considering fresh products with high perishability, or storable products (e.g., processed/treated) must be considered in order to avoid problems with transport or storability. A continuous supply without many fluctuations in properties of the feed is desired in order to develop sustainable and successful insect production.

Several companies today already focus on insect bioconversion of side streams, indicating that commercial insect production is a promising industry of high economic value; however, further optimizations are needed. Although several studies have been performed on the production of insects on low valuable biomass, additional research must explore diet requirements of the insects and growth and quality of insects produced on side streams. Focus here must be laid on valorising local, highly available side streams with acceptable processing costs.

## Figures and Tables

**Table 3:** Agricultural crop production data from FAOSTAT database [102] for 2018 and estimated availability of crop residues in tons for the EU(28). RTP-ratio is residue-to-product ratio, based on Ronzon et al. [101].

Agricultural Side Stream	Production	RTP-Ratio	Residue Production
Apples	13,849,593	0.20	2,769,919
Pears	2,582,274	0.20	516,455
Strawberries	1,271,750	0.20	254,350
Grapes	27,619,792	0.20	5,523,958
Oranges	6,515,370	0.20	1,303,074
**Fruit total**	**71,654,301**	**0.20**	**14,330,860**
Sugar beet	119,579,910	0.54	64,573,151
**Sugar crops total**	**121,860,062**	**0.61**	**74,334,638**
Chilies and pepper	2,588,420	0.20	517,684
Cabbages and other Brassicas	4,614,668	0.20	922,934
Cauliflowers and broccoli	2,387,488	0.20	477,498
Lettuce and chicory	2,842,937	0.20	568,587
Chicory roots	571,994	1.00	571,994
Cucumbers and gherkins	2,829,509	0.20	565,902
Eggplants	866,356	0.20	173,271
Leeks, other Alliaceous vegetables	752,681	0.20	150,536
Tomatoes	17,058,138	0.20	3,411,628
Lentils	115,340	0.20	23,068
Onions (dry)	5,942,422	0.20	1,188,484
Carrots	5,353,961	0.20	1,070,792
**Vegetables total**	**61,698,004**	**0.20**	**12,339,601**
Wheat	138,049,231	1.25	172,561,539
Barley	56,659,810	1.22	69,124,968
Triticale	9,845,068	1.39	13,635,419
Oats	7,785,890	1.18	9,187,350
Rye	6,236,406	1.18	7,358,959
Corn	2,725,683	1.54	4,197,552
Grain, mixed	3,131,853	1.39	4,337,616
**Cereals total**	**295,305,279**	**1.39**	**408,997,811**
Olives	13,778,370	1.60	21,976,500
Sunflower	10,003,030	1.86	18,605,636
Rapeseed	19,928,570	1.86	37,067,140
Soybean	2,912,120	1.36	3,960,483
**Oil crops total**	**47,980,763**	**1.60**	**76,529,317**
**Potatoes**	**52,253,108**	**1.00**	**52,253,108**

**Table 4:** Estimated number of poultry (×1000), porcine and cattle heads in the EU(28) in 2010 and 2018. Livestock represented as number of heads [102]. Variation represented as %.

Livestock	2010	2018	Variation
Porcine	256,799,114	261,647,761	1.89%
Cattle	29,020,397	26,850,232	−7.48%
Poultry	6,289,886	7,285,469	15.83%

**Table 5:** Tons of meat co-products and offal in the EU(28) in 2010 and 2018, based on [102,109].

Livestock	2010	2018
Porcine	5,135,982	5,232,955
Cattle	4,389,335	4,061,098
Poultry	471,741	546,410

**Table 6:** Animal co-products current uses: industrial products and pharmaceuticals [72].

Co-Product	Commercial Product
Bile	Detergent and pharmaceuticals
Bones	Adhesives, animal feed, calcium and phosphorous source (bone meal), glycerine, glue and collagen
Blood	Spray dried plasma, iron supplement, functional ingredient and fat replacer
Brains and spinal cords	Steroid, cholesterol, lecithin and cephalin
Fats and fatty acids	Biodegradable detergents, animal feed, biodiesel, cosmetics, lubricants, plasticisers, emulsifiers and solvents
Glands:	
Adrenal	Cortisone, epinephrine and norepinephrine
Liver	Heparin, vitamin B12, pet food and bile (detergent and pharmaceuticals)
Pancreas	Chymotrypsin, insulin, pancreatin, trypsin and glucagon
Pituitary glands	ACTH and prolactin
Spleen	Ferritin
Thymus	Thymosin
Thyroid	TSH, hormones and so on
Kidney	Pet food
Hides and skins	Gelatin, collagen based adhesives and leather
Hairs, wool, skins, feathers, nails, horns and hooves	Fibers, collagen and glue
Hearts	Pet food
Intestines	Sausage casings, strings, heparin and small intestinal sub mucosa materials for clinical applications
Lungs	Pet food and heparin
Ovaries	Estrogen
Stomach and tripe	Pet food, glue, pepsin, rennin, lipase and trypsin
Trachea	Chondroitin sulphate

## Data Availability

Not applicable.

## References

[1] Walston J.D. (2012). Sarcopenia in older adults. Curr. Opin. Rheumatol..

[2] United Nations Department of Economic and Social Affairs (2019). World Population Prospects 2019: Highlights.

[3] Pelletier N., Tyedmers P. (2010). Forecasting potential global environmental costs of livestock production 2000–2050. Proc. Natl. Acad. Sci. USA.

[4] FAO (2017). The Future of Food and Agriculture: Trends and Challenges.

[5] FAO (2011). Global Food Losses and Food Waste—Extent, Causes and Prevention.

[6] Stenmarck Å., Jensen C., Quested T., Moates G., Buksti M., Cseh B., Juul S., Parry A., Politano A., Redlingshofer B. (2016). Estimates of European Food Waste Levels.

[7] EFFPA Figures & Network. https://www.effpa.eu/figures-network/.

[8] Godfray H.C.J., Pretty J., Thomas S.M., Warham E.J., Beddington J.R. (2011). Linking policy on climate and food. Science.

[9] Gustavsson J., Bos-Brouwers H., Timmermans T., Hansen O.-J., Møller H., Anderson G., O’connor C., Soethoudt H., Quested T., Easteal S. (2014). FUSIONS Definitional Framework for Food Waste.

[10] Diener S., Zurbrügg C., Tockner K. (2009). Conversion of organic material by black soldier fly larvae: Establishing optimal feeding rates. Waste Manag. Res..

[11] Van Broekhoven S., Oonincx D.G.A.B., van Huis A., van Loon J.J.A. (2015). Growth performance and feed conversion efficiency of three edible mealworm species (Coleoptera: Tenebrionidae) on diets composed of organic by-products. J. Insect Physiol..

[12] Oonincx D.G.A.B., Van Broekhoven S., Van Huis A., Van Loon J.J.A. (2015). Feed conversion, survival and development, and composition of four insect species on diets composed of food by-products. PLoS ONE.

[13] Bessa L.W., Pieterse E., Sigge G., Hoffman L.C. (2017). Insects as Human Food; From Farm to Fork. J. Sci. Food Agric..

[14] Van Huis A. (2013). Potential of insects as food and feed in assuring food security. Annu. Rev. Entomol..

[15] Benno Meyer-Rochow V. (1975). Can insects help to ease the problem of world food shortage?. Search.

[16] Oonincx D.G.A.B., van Itterbeeck J., Heetkamp M.J.W., van den Brand H., van Loon J.J.A., van Huis A. (2010). An exploration on greenhouse gas and ammonia production by insect species suitable for animal or human consumption. PLoS ONE.

[17] Bjørge J.D., Overgaard J., Malte H., Gianotten N., Heckmann L.H. (2018). Role of temperature on growth and metabolic rate in the tenebrionid beetles *Alphitobius diaperinus* and *Tenebrio molitor*. J. Insect Physiol..

[18] Oonincx D.G.A.B., de Boer I.J.M. (2012). Environmental Impact of the Production of Mealworms as a Protein Source for Humans—A Life Cycle Assessment. PLoS ONE.

[19] Miglietta P.P., De Leo F., Ruberti M., Massari S. (2015). Mealworms for food: A water footprint perspective. Water.

[20] Rumpold B.A., Schlüter O.K. (2013). Potential and challenges of insects as an innovative source for food and feed production. Innov. Food Sci. Emerg. Technol..

[21] Surendra K.C., Tomberlin J.K., van Huis A., Cammack J.A., Heckmann L.H.L., Khanal S.K. (2020). Rethinking organic wastes bioconversion: Evaluating the potential of the black soldier fly (*Hermetia illucens* (L.)) (Diptera: Stratiomyidae) (BSF). Waste Manag..

[22] Turck D., Castenmiller J., De Henauw S., Hirsch-Ernst K.I., Kearney J., Maciuk A., Mangelsdorf I., McArdle H.J., Naska A., Pelaez C. (2021). Safety of dried yellow mealworm (*Tenebrio molitor* larva) as a novel food pursuant to Regulation (EU) 2015/2283. EFSA J..

[23] Turck D., Castenmiller J., De Henauw S., Hirsch-Ernst K.I., Kearney J., Maciuk A., Mangelsdorf I., McArdle H.J., Naska A., Pelaez C. (2021). Safety of frozen and dried formulations from migratory locust (*Locusta migratoria*) as a Novel food pursuant to Regulation (EU) 2015/2283. EFSA J..

[24] Montanari F., Pinto de Moura A., Miguel Cunha L. (2021). The EU Regulatory Framework for Insects as Food and Feed and Its Current Constraints. Production and Commercialization of Insects as Food and Feed.

[25] Rumpold B.A., Schlüter O.K. (2013). Nutritional composition and safety aspects of edible insects. Mol. Nutr. Food Res..

[26] Zhang X., Tang H., Chen G., Qiao L., Li J., Liu B., Liu Z., Li M., Liu X. (2019). Growth performance and nutritional profile of mealworms reared on corn stover, soybean meal, and distillers’ grains. Eur. Food Res. Technol..

[27] Li L., Zhao Z., Liu H. (2013). Feasibility of feeding yellow mealworm (*Tenebrio molitor* L.) in bioregenerative life support systems as a source of animal protein for humans. Acta Astronaut..

[28] Law Y., Wein L. (2018). Reversing the nutrient drain through urban insect farming—Opportunities and challenges. AIMS Bioeng..

[29] Ojha S., Bußler S., Schlüter O.K. (2020). Food waste valorisation and circular economy concepts in insect production and processing. Waste Manag..

[30] Poveda J. (2021). Insect frass in the development of sustainable agriculture. A review. Agron. Sustain. Dev..

[31] Robinson W.H. (2005). Urban Insects and Arachnids—A Handbook of Urban Entomology.

[32] Meireles E.A., Carneiro C.N.B., DaMatta R.A., Samuels R.I., Silva C.P. (2009). Digestion of starch granules from maize, potato and wheat by larvae of the the yellow mealworm, *Tenebrio molitor* and the Mexican bean weevil, Zabrotes subfasciatus. J. Insect Sci..

[33] Applebaum S.W. (1966). Digestion of potato starch by larvae of the flour beetle, Tribolium castaneum. J. Nutr..

[34] Po E., Sinha N.K. (2011). Potatoes: Production, Quality, and Major Processed Products. Handbook of Vegetables and Vegetable Processing.

[35] Nenaah G. (2011). Individual and synergistic toxicity of solanaceous glycoalkaloids against two coleopteran stored-product insects. J. Pest Sci..

[36] Ventrella E., Marciniak P., Adamski Z., Rosiński G., Chowański S., Falabella P., Scrano L., Bufo S.A. (2015). Cardioactive properties of Solanaceae plant extracts and pure glycoalkaloids on Zophobas atratus. Insect Sci..

[37] Ramos-Elorduy J., González E.A., Hernández A.R., Pino J.M. (2002). Use of *Tenebrio molitor* (Coleoptera: Tenebrionidae) to recycle organic wastes and as feed for broiler chickens. J. Econ. Entomol..

[38] Stull V.J., Kersten M., Bergmans R.S., Patz J.A., Paskewitz S. (2019). Crude Protein, Amino Acid, and Iron Content of *Tenebrio molitor* (Coleoptera, Tenebrionidae) Reared on an Agricultural Byproduct from Maize Production: An Exploratory Study. Ann. Entomol. Soc. Am..

[39] Mancini S., Fratini F., Turchi B., Mattioli S., Dal Bosco A., Tuccinardi T., Nozic S., Paci G. (2019). Former foodstuff products in *Tenebrio molitor* rearing: Effects on growth, chemical composition, microbiological load, and antioxidant status. Animals.

[40] Harsányi E., Juhász C., Kovács E., Huzsvai L., Pintér R., Fekete G., Varga Z.I., Aleksza L., Gyuricza C. (2020). Evaluation of organic wastes as substrates for rearing zophobas morio, *Tenebrio molitor*, and *Acheta domesticus* larvae as alternative feed supplements. Insects.

[41] Peng X., Chen H. (2008). Single cell oil production in solid-state fermentation by Microsphaeropsis sp. from steam-exploded wheat straw mixed with wheat bran. Bioresour. Technol..

[42] House H.L. (1965). Effects of Low Levels of the Nutrient Content of a Food and of Nutrient Imbalance on the Feeding and the Nutrition of a Phytophagous Larva, *Celerio euphorbiae* (Linnaeus) (Lepidoptera: Sphingidae). Can. Entomol..

[43] Morales-Ramos J.A., Rojas M.G., Shapiro-Llan D.I., Tedders W.L. (2013). Use of nutrient self-selection as a diet refining tool in *Tenebrio molitor* (Coleoptera: Tenebrionidae). J. Entomol. Sci..

[44] Melis R., Braca A., Sanna R., Spada S., Mulas G., Fadda M.L., Sassu M.M., Serra G., Anedda R. (2019). Metabolic response of yellow mealworm larvae to two alternative rearing substrates. Metabolomics.

[45] Ruschioni S., Loreto N., Foligni R., Mannozzi C., Raffaelli N., Zamporlini F., Pasquini M., Roncolini A., Cardinali F., Osimani A. (2020). Addition of olive pomace to feeding substrate affects growth performance and nutritional value of mealworm (*Tenebrio molitor* L.) larvae. Foods.

[46] Coudron C., Sprangher T., Elliot D., Halstead J. (2019). Insect Breeding: Lab Scale and Pilot Scale Experiments with Mealworm and Black Soldier Fly.

[47] Cornelis Y. (2019). Verhakselde witloofwortels, een lekkernij voor meelwormen. Proeftuinnieuws.

[48] Wadhwa M., Bakshi M.P.S. (2013). Utilization of Fruit and Vegetable Wastes as Livestock Feed and as Substrates for Generation of Other Value-Added Products.

[49] Mussoline W., Esposito G., Giordano A., Lens P. (2013). The Anaerobic Digestion of Rice Straw: A Review. Crit. Rev. Environ. Sci. Technol..

[50] Yang S.S., Chen Y.D., Zhang Y., Zhou H.M., Ji X.Y., He L., Xing D.F., Ren N.Q., Ho S.H., Wu W.M. (2019). A novel clean production approach to utilize crop waste residues as co-diet for mealworm (*Tenebrio molitor*) biomass production with biochar as byproduct for heavy metal removal. Environ. Pollut..

[51] Weissman D.B., Rentz D.C.F. (1977). Feral house crickets *Acheta domesticus* (L.) (Orthoptera:Gryllidae) in Southern California. Entomol. News.

[52] Ghouri A.S.K. (1961). Home and Distribution of the House Cricket *Acheta domesticus* L.. Nature.

[53] Clifford C.W., Woodring J.P. (1990). Methods for rearing the house cricket, *Acheta domesticus* (L.), along with baseline values for feeding rates, growth rates, development times, and blood composition. J. Appl. Entomol..

[54] Collavo A., Glew R.H., Huang Y.S., Chuang L.T., Bosse R., Paoletti M.G., Paoletti M.G. (2005). House Cricket Small-scale Farming. Ecological Implications of Minilivestock: Potential of Insects, Rodents, Frogs and Snails.

[55] Sorjonen J.M., Valtonen A., Hirvisalo E., Karhapää M., Lehtovaara V.J., Lindgren J., Marnila P., Mooney P., Mäki M., Siljander-Rasi H. (2019). The plant-based by-product diets for the mass-rearing of *Acheta domesticus* and *Gryllus bimaculatus*. PLoS ONE.

[56] Patton R.L. (1967). Oligidic Diets for *Acheta domesticus* (Orthoptera: Gryllidae). Ann. Entomol. Soc. Am..

[57] Lundy M.E., Parrella M.P. (2015). Crickets are not a free lunch: Protein capture from scalable organic side-streams via high-density populations of *Acheta domesticus*. PLoS ONE.

[58] Song P., Zheng X., Li Y., Zhang K., Huang J., Li H., Zhang H., Liu L., Wei C., Mansaray L.R. (2020). Estimating reed loss caused by *Locusta migratoria* manilensis using UAV-based hyperspectral data. Sci. Total Environ..

[59] Waloff Z. (1940). The Distribution and Migrations of Locusta in Europe. Bull. Entomol. Res..

[60] Raubenheimer D., Simpson S.J. (2003). Nutrient balancing in grasshoppers: Behavioural and physiological correlates of dietary breadth. J. Exp. Biol..

[61] Scanlan J.C., Grant W.E., Hunter D.M., Milner R.J. (2001). Habitat and environmental factors influencing the control of migratory locusts (*Locusta migratoria*) with an entomopathogenic fungus (*Metarhizium anisopliae*). Ecol. Modell..

[62] Dadd R.H. (1960). The nutritional requirements of locusts-I Development of synthetic diets and lipid requirements. J. Insect Physiol..

[63] Dadd R.H. (1960). Observations on the palatability and utilisation of food by locusts, with particular reference to the interpretation of performances in growth trials using synthetic diets. Entomol. Exp. Appl..

[64] Dadd R.H. (1960). The nutritional requirements of locusts—III carbohydrate requirements and utilization. J. Insect Physiol..

[65] Dadd R.H. (1961). The nutritional requirements of locusts—V: Observations on essential fatty acids, chlorophyll, nutritional salt mixtures, and the protein or amino acid components of synthetic diets. J. Insect Physiol..

[66] Dadd R.H. (1961). The nutritional requirements of locusts—IV. Requirements for vitamins of the B complex. J. Insect Physiol..

[67] Mehrotra K.N., Rao P.J., Farooqi T.N.A. (1972). The consumption, digestion and utilization of food by locusts. Entomol. Exp. Appl..

[68] Bernays E.A., Chapman R.F., Macdonald J., Salter J.E.R. (1976). The degree of oligophagy in *Locusta migratoria* (L.). Ecol. Entomol..

[69] Blunt D.L., Wilkinson H., Edwards D.C. (1931). Report on the 1931 Locust Invasion of Kenya.

[70] Beenakkers A.M.T., Meisen M.A.H.Q., Scheres J.M.J.C. (1971). Influence of temperature and food on growth and digestion in fifth instar larvae and adults of Locusta. J. Insect Physiol..

[71] Kwak K.W., Kim S.Y., An K.S., Kim Y.S., Park K., Kim E., Hwang J.S., Kim M.A., Ryu H.Y., Yoon H.J. (2020). Subacute Oral Toxicity Evaluation of Freeze-Dried Powder of *Locusta migratoria*. Food Sci. Anim. Resour..

[72] Lynch S.A., Mullen A.M., O’Neill E., Drummond L., Álvarez C. (2018). Opportunities and perspectives for utilisation of co-products in the meat industry. Meat Sci..

[73] Mullen A.M., Álvarez C., Pojić M., Hadnadev T.D., Papageorgiou M. (2015). Chapter 2—Classification and target compounds. Food Waste Recovery.

[74] Woodring J.P., Clifford C.W., Beckman B.R. (1979). Food utilization and metabolic efficiency in larval and adult house crickets. J. Insect Physiol..

[75] Davis G.R.F. (1975). Essential Dietary Amino Acids for Growth of Larvae of the Yellow Mealworm. J. Nutr..

[76] House H.L. (1969). Effects of different proportions of nutrients on insects. Entomol. Exp. Appl..

[77] Kraus S., Monchanin C., Gomez-Moracho T., Lihoreau M. (2019). Insect Diet. Encyclopedia of Animal Cognition and Behavior.

[78] Kips L., Van Droogenbroeck B. (2014). Valorisatie Van Groente- en Fruitreststromen: Opportuniteiten en Knelpunten.

[79] IPIFF EU Legislation. https://ipiff.org/insects-eu-legislation/.

[80] Bos-Brouwers H.E., Langelaan H.C., Sanders J.P., van Dijk M., van Vuuren A. (2012). Chances for Biomass: Integrated Valorisation of Biomass Resources.

[81] EFFPA Reducing Food Waste. https://www.effpa.eu/reducing-food-waste/.

[82] Smetana S., Palanisamy M., Mathys A., Heinz V. (2016). Sustainability of insect use for feed and food: Life Cycle Assessment perspective. J. Clean. Prod..

[83] Liu C., Masri J., Perez V., Maya C., Zhao J. (2020). Growth performance and nutrient composition of mealworms (*Tenebrio molitor*) fed on fresh plant materials-supplemented diets. Foods.

[84] Ribeiro N., Abelho M., Costa R. (2018). A Review of the Scientific Literature for Optimal Conditions for Mass Rearing *Tenebrio molitor* (Coleoptera: Tenebrionidae). J. Entomol. Sci..

[85] Davis G.R., Sosulski F.W. (1974). Nutritional quality of oilseed protein isolates as determined with larvae of the yellow mealworm, *Tenebrio molitor* L.. J. Nutr..

[86] Morales-Ramos J.A., Rojas M.G., Dossey A.T., Berhow M. (2020). Self-selection of food ingredients and agricultural by-products by the house cricket, *Acheta domesticus* (Orthoptera: Gryllidae): A holistic approach to develop optimized diets. PLoS ONE.

[87] Hinks C.F., Erlandson M.A. (1994). Rearing Grasshoppers and Locusts: Review, Rationale and Update. J. Orthoptera Res..

[88] Mulkern G.B. (1969). Behavioral influences on food selection in grasshoppers (Orthoptera: Acrididae). Entomol. Exp. Appl..

[89] Gangwere S.K. (1960). Notes on drinking and the need for water in Orthoptera. Can. Entomol..

[90] Veenenbos M.E., Oonincx D.G.A.B. (2017). Carrot supplementation does not affect house cricket performance (*Acheta domesticus*). J. Insects Food Feed.

[91] Benno Meyer-Rochow V., Gahukar R.T., Ghosh S., Jung C., Smith J. (2021). Foods Chemical Composition, Nutrient Quality and Acceptability of Edible Insects Are Affected by Species, Developmental Stage, Gender, Diet, and Processing Method. Foods.

[92] Melgar-Lalanne G., Hernandez-Alvarez A.J., Salinas-Castro A. (2019). Edible insects processing: Traditional and innovative technologies. Compr. Rev. Food Sci. Food Saf..

[93] Kröncke N., Grebenteuch S., Keil C., Demtröder S., Kroh L., Thünemann A.F., Benning R., Haase H. (2019). Effect of different drying methods on nutrient quality of the yellow mealworm (*Tenebrio molitor* L.). Insects.

[94] Lenaerts S., Van Der Borght M., Callens A., Van Campenhout L. (2018). Suitability of microwave drying for mealworms (*Tenebrio molitor*) as alternative to freeze drying: Impact on nutritional quality and colour. Food Chem..

[95] Bawa M., Songsermpong S., Kaewtapee C., Chanput W. (2020). Effect of Diet on the Growth Performance, Feed Conversion, and Nutrient Content of the House Cricket. J. Insect Sci..

[96] Oonincx D.G.A.B., Van Der Poel A.F.B. (2011). Effects of diet on the chemical composition of migratory locusts (*Locusta migratoria*). Zoo Biol..

[97] Corrado S., Sala S. (2018). Food waste accounting along global and European food supply chains: State of the art and outlook. Waste Manag..

[98] Caldeira C., De Laurentiis V., Corrado S., van Holsteijn F., Sala S. (2019). Quantification of food waste per product group along the food supply chain in the European Union: A mass flow analysis. Resour. Conserv. Recycl..

[99] Lal R. (2005). World crop residues production and implications of its use as a biofuel. Environ. Int..

[100] Searle S., Malins C. (2013). Availability of Cellulosic Residues and Wastes in the EU.

[101] Ronzon T., Piotrowski S., Carus M. (2015). DataM—Biomass Estimates (v3): A New Database to Quantify Biomass Availability in the European Union.

[102] FAO FAOSTAT. http://www.fao.org/faostat/en/#home.

[103] Wirsenius S. (2000). Human Use of Land and Organic Materials: Modeling the Turnover of Biomass in the Global Food System.

[104] Pinotti L., Giromini C., Ottoboni M., Tretola M., Marchis D. (2019). Insects and former foodstuffs for upgrading food waste biomasses/streams to feed ingredients for farm animals. Animal.

[105] Searle S.Y., Malins C.J. (2016). Waste and residue availability for advanced biofuel production in EU Member States. Biomass Bioenergy.

[106] Kemna R., Holsteijn F.V., Lee P., Sims E. (2017). Optimal Food Storage Conditions in Refrigeration Appliances.

[107] Jackson A., Newton R. (2016). Project to Model the Use of Fisheries By-Products in the Production of Marine Ingredients, with Special Reference the Omega 3 Fatty Acids EPA and DHA.

[108] Pearson A.M., Dutson T.R. (2013). Inedible Meat By-Products.

[109] MLC Services Angiestuff. http://www.angiestuff.com/ADNet/index_htm_files/3a.WalshMLCAdnet.pdf.

[110] Gac A., Lapasin C., Laspière P.T., Guardia S., Ponchant P., Chevillon P., Nassy G. (2014). Co-Products from Meat Processing: The Allocation Issue.

[111] Chahine M., de Haro Marti M.E., Matuk C., Aris A., Campbell J., Polo J., Bach A. (2019). Effects of spray-dried plasma protein in diets of early lactation dairy cows on health, milking and reproductive performance. Anim. Feed Sci. Technol..

[112] Tapia-Paniagua S.T., Balebona M.D.C., Firmino J.P., Rodríguez C., Polo J., Moriñigo M.A., Gisbert E. (2020). The effect of spray-dried porcine plasma on gilthead seabream (*Sparus aurata*) intestinal microbiota. Aquac. Nutr..

[113] Zhang R.J., Lee B., Chang H.H. (2019). What Is Missing in Food Loss and Waste Analyses? A Close Look at Fruit and Vegetable Wholesale Markets. Sustainability.

[114] Roels K., van Gijseghem D. (2017). The Impact of Cosmetic Quality Standards on Food Losses in the Flemish Fruit and Vegetable Sector: Summary Report.

[115] Flemish Food Supply Chain Platform for Food Loss (2017). Food Waste and Food Losses: Prevention and Valorisation.

[116] Van Verbond B.T. (2019). Overview of Received Products and Their Destination for Belgian Fruit and Vegetable Auctions.

[117] Rentizelas A.A., Tolis A.J., Tatsiopoulos I.P. (2009). Logistics issues of biomass: The storage problem and the multi-biomass supply chain. Renew. Sustain. Energy Rev..

[118] Skoulou V., Zabaniotou A. (2007). Investigation of agricultural and animal wastes in Greece and their allocation to potential application for energy production. Renew. Sustain. Energy Rev..

[119] Allen J., Browne M., Hunter A., Boyd J., Palmer H. (1998). Logistics management and costs of biomass fuel supply. Int. J. Phys. Distrib. Logist. Manag..

[120] Straub P., Tanga C.M., Osuga I., Windisch W., Subramanian S. (2019). Experimental feeding studies with crickets and locusts on the use of feed mixtures composed of storable feed materials commonly used in livestock production. Anim. Feed Sci. Technol..

[121] Louveaux A., Mainguet A.M., Gillon Y. (1980). Feeding Locusts on Freeze-Dried Plants: A New Rearing Method for Herbivorous Insects. Entomol. Exp. Appl..

[122] FEFAC (2019). Annual Report 2018–2019.

[123] Álvarez C., Mullen A.M., Pojić M., Hadnađev T.D., Papageorgiou M. (2021). Classification and target compounds. Food Waste Recovery.

[124] Cheng J.Y., Chiu S.L., Lo I.M. (2017). Effects of moisture content of food waste on residue separation, larval growth and larval survival in black soldier fly bioconversion. Waste Manag..

[125] Fowles T.M., Nansen C., Närvänen E., Mesiranta N., Mattila M., Heikkinen A. (2020). Insect-Based Bioconversion: Value from Food Waste. Cham. Food Waste Management.

[126] Zhan S., Fang G., Cai M., Kou Z., Xu J., Cao Y., Bai L., Zhang Y., Jiang Y., Luo X. (2020). Genomic landscape and genetic manipulation of the black soldier fly *Hermetia illucens*, a natural waste recycler. Cell Res..

[127] Rumbos C.I., Adamaki-Sotiraki C., Gourgouta M., Karapanagiotidis I.T., Asimaki A., Mente E., Athanassiou C.G. (2021). Strain matters: Strain effect on the larval growth and performance of the yellow mealworm, *Tenebrio molitor* L.. J. Insects Food Feed.

[128] Chieco C., Morrone L., Bertazza G., Cappellozza S., Saviane A., Gai F., Di Virgilio N., Rossi F. (2019). The Effect of Strain and Rearing Medium on the Chemical Composition, Fatty Acid Profile and Carotenoid Content in Silkworm (Bombyx mori) Pupae. Animals.

[129] Jensen K., Kristensen T.N., Heckmann L.L., Sørensen J.G., van Huis A., Tomberlin J.K. (2017). Breeding and maintaining high-quality insects Insects as food and feed. Insects as Food and Feed: From Production to Consumption.

[130] Kaya C., Generalovic T.N., Ståhls G., Hauser M., Samayoa A.C., Nunes-Silva C.G., Roxburgh H., Wohlfahrt J., Ewusie E.A., Kenis M. (2021). Global population genetic structure and demographic trajectories of the black soldier fly, *Hermetia illucens*. BMC Biol..

[131] Gupta S., Lee J.J.L., Chen W.N. (2018). Analysis of improved nutritional composition of potential functional food (Okara) after probiotic solid-state fermentation. J. Agric. Food Chem..

[132] Lievens S., Poma G., De Smet J., Van Campenhout L., Covaci A., Van Der Borght M. (2021). Chemical safety of black soldier fly larvae (*Hermetia illucens* ), knowledge gaps and recommendations for future research: A critical review. J. Insects Food Feed.

[133] Wynants E., Frooninckx L., Crauwels S., Verreth C., De Smet J., Sandrock C., Wohlfart J., Van Schelt J., Depraetere S., Lievens B. (2019). Assessing the Microbiota of Black Soldier Fly Larvae (*Hermetia illucens*) Reared on Organic Waste Streams on Four Different Locations at Laboratory and Large Scale. Microb. Ecol..

[134] EAAP. https://www.eaap.org/study-commissions/insects/.

[135] Bosch G., Oonincx D.G.A.B., Jordan H.R., Zhang J., van Loon J.J.A., van Huis A., Tomberlin J.K. (2020). Standardisation of quantitative resource conversion studies with black soldier fly larvae. J. Insects Food Feed.

